# Imidazolium Triflate Ionic Liquids’ Capacitance–Potential Relationships and Transport Properties Affected by Cation Chain Lengths

**DOI:** 10.1021/acsmeasuresciau.1c00015

**Published:** 2021-08-10

**Authors:** Niroodha R. Pitawela, Scott K. Shaw

**Affiliations:** Department of Chemistry, University of Iowa, Iowa City, Iowa 52242, United States

**Keywords:** ionic liquids, capacitance, voltage, alkyl chain length, hysteresis, energy storage

## Abstract

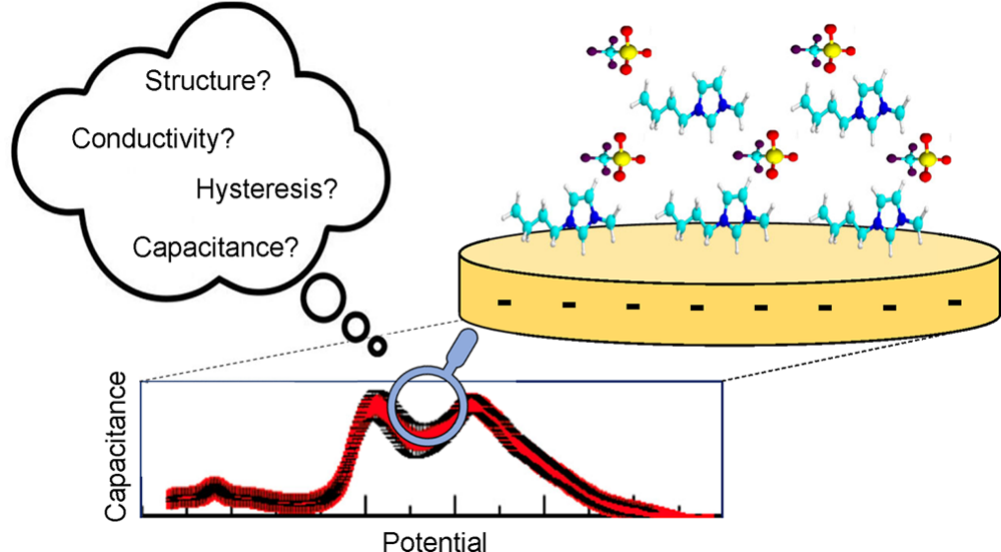

In this paper we report the effects of five imidazolium cations with varying alkyl chain lengths to study the effects of cation size on capacitance versus voltage behavior. The cations include ethyl-, butyl-, hexyl-, octyl-, and decyl-3-methylimidazolium, all paired with a triflate anion. We analyze the capacitance with respect to the cation alkyl chain length qualitatively and quantitatively by analyzing changes in the capacitance–potential curvature shape and magnitude across several standard scanning protocols and electrochemical techniques. Further, three transport properties (viscosity, diffusion coefficient, and electrical conductivity) are experimentally determined and integrated into the outcomes. Ultimately, we find higher viscosities, lower diffusion coefficients, and lower electrical conductivities when the alkyl chain length is increased. Also, capacitance values increase with cation size, except 1-octyl-3-methylimidazolium, which does not follow an otherwise linear trend. This capacitive increase is most pronounced when sweeping the potential in the cathodic direction. These findings challenge the conventional hypothesis that increasing the length of the alkyl chain of imidazolium cations diminishes the capacitance and ionic liquid performance in charge storage.

## 1 Introduction

Environmentally sustainable energy resources such as wind, solar, and hydropower are projected to double production within the next five decades.^1^ To effectively use this renewable resource, new modular and grid-scale storage technologies are needed.^1,2^ Electrochemical double-layer capacitors (EDLCs) store energy in the form of an electrical charge at the electrode–solution interface in response to a perturbation of the electrochemical potential.^3^ They have attracted significant attention as promising electrochemical energy storage systems due to their high power densities (∼10 kW/kg, 1 order of magnitude greater than that of lithium ion batteries)^2,4,5^ and very long life cycles (>100 000 charge–discharge cycles).^4,5^ Currently, EDLCs are used to power backup memory in computers, hybrid electric vehicles, and other energy storage systems not connected to conventional power grids.^6^

The processes of energy storage and release in EDLCs are linked to the structure of an electrochemical double layer (EDL) that develops when the system is charged. The EDL and its formation/disruption kinetics dominate supercapacitors’ energy and power density characteristics.^8^ Most EDLCs in the market use aprotic solvents such as acetonitrile and propylene carbonate.^9,10^ However, solvent flammability poses safety issues, and the electrochemical stability of EDLCs decreases with increasing temperatures, resulting in a significant device cycle life degradation.^10,11^

Ionic liquids (ILs) are promising replacements for traditional electrolytes due to their ability to operate at wide voltage windows (approaching 6 V), which would increase the energy density of these devices via eq 1(12−14)
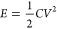
1where *E* = energy, *C* = capacitance, and *V* = voltage; thus, increases in the applied voltage have an exponential impact on the theoretical energy density. While ILs can be toxic^15^ and are currently more expensive than traditional solvents (e.g., water), they are nonvolatile and nonflammable allowing their safe usage at temperatures well over 150 °C.^16−18^ ILs’ ionic properties and electrochemical behaviors can be thoughtfully tuned through a prudent selection of cation–anion pairings, and their performance for energy storage can be optimized.^19^ While there are numerous ways of combining ILs, existing investigations are focused on a few IL classes, with single anion–cation pairs (as opposed to ternary mixtures).^20^ For example, 1-alkyl-3-methylimidazolium cation-based ILs have received considerable attention as promising electrolytes in EDLCs due to a relatively low viscosity (52 mPa·s at 20 °C), greater conductivity (i.e., 0.5–13 mS/cm), and lower hydroscopicity.^21^ Likewise, in recent years, trifluoromethanesulfonate [TFO]^−^ anion-based ILs have drawn significant attention in the IL community, as the [TFO]^−^ anion is hydrolytically stable, which makes it preferable over hydrolytically unstable [PF_6_]^−^ or [BF_4_]^−^ anions.^22^

Several previous studies have reported on the imidazolium cation-based ILs’ alkyl chain length dependence on capacitance.^20,23−28^ Lockett and co-workers report the effect of the cation chain length of imidazolium chloride-based ILs on a glassy carbon electrode. They concluded that longer alkyl chains result in a lower measured capacitance, likely as a result of the lower permittivity.^26^ A similar trend has been observed for imidazolium tetrafluoroborate-based ILs on a gold electrode.^27^ Liu et al.^25^ and Jung et al.^29^ also observed a lower capacitance for ILs with longer alkyl chain lengths. However, Roling and co-workers observed an increased capacitance for longer alkyl chain lengths of imidazolium cations on a Au(111) electrode surface and suggest that, apart from the cation size, other factors such as cation flexibility and polarizability play important roles for capacitance.^28^ Also, recent studies performed by Aken et al.^30^ and Wu and co-workers^20^ demonstrated that the capacitance can be increased by increasing the cation chain length from ethyl (*n* = 2) to hexyl (*n* = 6). These contradictory prior results based on the cation chain length effect on capacitance highlight the necessity of providing further solid experimental basis of the same, particularly across a broad series of ILs and with multiple data acquisition methods, as we report here.

In the EDLC design, both interfacial and bulk properties must be considered in order to provide a complete understanding of (1) ion movements in bulk electrolyte and (2) the reorganization processes occurring at the interface.^31^ Hence, we explore changes in three transport properties of these pure imidazolium ILs as a function of cation chain length: viscosity, electrical conductivity, and diffusion. All these properties describe the motion of ions at the molecular level and are related through Nernst–Einstein (conductivity and diffusion) and Stokes–Einstein (SE) (diffusion and viscosity) equations.^32^

In ILs, mass transport is well-studied, through viscosity measurements. Viscosity describes the hydrodynamics of the system, such as resistance to flow. However, there are purity-related discrepancies among existing data reported for individual ILs.^33−35^ Therefore, we analyze the purity of the IL samples using ^1^H NMR experiments to identify possible impurities. Furthermore, while diffusion in ILs leads to conductivity and viscosity, the mode of diffusion is less well understood. Particularly, self-diffusion (free ion) and mutual diffusion (ion pairs) of ILs may both be possible and would have a significant impact on the physical properties of the IL system.^36^ Hence, also we employ pulsed-field-gradient spin–echo (PFGSE) (also known as diffusion-ordered spectroscopy (DOSY)) NMR to determine the self-diffusion coefficients of the ILs examined here.^37,38^ Conductivity is a critical factor in characterizing IL electrochemical systems, and it is directly related to the number and mobility of available charge carriers.^36^ Previous studies have determined the conductivity of imidazolium-based ILs; the electrical conductivity of pure ILs is generally at least 1 order of magnitude lower than that of aqueous solvent systems and aqueous solvent-IL mixtures.^39,40^ Some ILs exhibit much lower conductivity than others, even with comparable viscosities.^41^ The low conductivity values of ILs have been attributed to a limited number of available charge carriers due to ion pairing and/or ion aggregation and to the reduced ion mobility due to larger ion sizes.^42^ The ionic character of particular ion dense ILs has strongly influenced the development of electrochemical devices such as EDLCs,^43−45^ fuel cells,^46^ and batteries.^47−49^^36,50^

**Figure 1:**
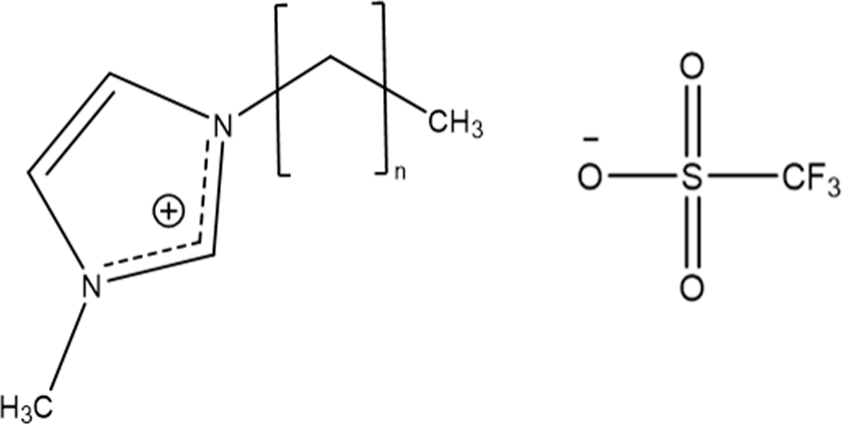
Structure of the ions constituting the ILs used in this study, namely, C_*n*_-3-methylimidazolium trifluoromethanesulfonate/triflate, where *n* = 1, 3, 5, 7, and 9 corresponding to [Emim]^+^, [Bmim]^+^, [Hmim]^+^, [Omim]^+^, and [Dmim]^+^ cations.

In this work, we explore a series of *n*-alkyl-3-methylimidazolium trifluoromethanesulfonate [n-mim][TFO]-based ILs (Figure 1) as a systematic series of ionic liquids for capacitive energy storage. Thereby, we investigate the impact of molecular structure on the capacitance at the electrode-IL interface for ILs containing different imidazolium cations with varying tail lengths based on capacitance–potential relationships. Our examination includes single-frequency impedance and linear alternating current (AC) voltammetry to acquire impedance data to examine the profile of electrochemical capacitance as it is influenced by the cation chain length. The central motivation of this study is to clarify the influence of the alkyl chain length of imidazolium cations on the EDL capacitance. Moreover, we measure the conductivity and viscosity of these IL samples. Then the relationship between these transport properties is presented in a Walden plot to qualitatively characterize the ionic association in ILs in comparison to a KCl solution that is fully dissociated.^51^ Diffusion coefficients obtained from DOSY NMR measurements are then compared to the conductivity and viscosity measurements and qualitative results obtained with the Walden plot approach.

## 2 Experimental Section

### 2.1 Materials and Methods

1-Ethyl-3-methylimidazolium trifluoromethanesulfonate [Emim][TFO], 1-butyl-3-methylimidazolium trifluoromethanesulfonate [Bmim][TFO], 1-hexyl-3-methylimidazolium trifluoromethanesulfonate [Hmim][TFO], 1-methyl-3-octylimidazolium trifluoromethanesulfonate [Omim][TFO], and 1-decyl-3-methylimidazolium trifluoromethanesulfonate [Dmim][TFO] ≥99% were acquired from Iolitec Ionic Liquids Technologies, GmbH. The ILs are placed under vacuum at 60 °C for several days (e.g., 4 d) for further drying. The residual water content in each IL is determined in triplicate using an 831 coulometric Karl Fischer (KF) titrator with a 300 μL IL volume. The water content of each IL is given in the Supporting Information under SI 1.

The purity of the ionic liquids was analyzed by performing solution ^1^H NMR experiments. The samples are prepared by dissolving ∼60 μL of IL in 540 μL of a deuterated solvent (i.e., acetonitrile-*d*_3_ (Cambridge Isotopic Laboratories, 99.8% purity)) via a sonication for 15 min. The samples are sonicated again for ∼30 min just before the NMR measurements. The solution-phase ^1^H NMR experiments are performed using an Avance Bruker NMR spectrometer operated at 500 MHz, and this NMR instrument uses a 5 mm PABBO BB-1H/D Z-GRD probe. All the ^1^H chemical shifts are referenced to the broad singlet peak of the residual solvent proton (HDO) centered at 1.94 ppm, and the chemical shifts are reported in parts per million downfield from tetramethylsilane (TMS), unless mentioned otherwise. Topspin software ver. 2.1 is used in the data analysis.

The three-electrode system with a 2 mL (total volume) conical glass vial including a custom-built airtight polytetrafluoroethylene (PTFE) cap serves as the electrochemical cell. The electrochemical cell is cleaned with Nochromix acid (ammonium persulfate in 98% H_2_SO_4_) (>6 h), rinsed with copious amounts of ultrapure water (Milli-Q system, 18.2 MΩ cm), soaked in 3 M nitric acid (>6 h), rinsed with copious amounts of deionized (DI) water, rinsed again with boiling ultrapure water, and finally dried in an ambient-pressure oven at 120 °C before being introduced into a nitrogen glovebox (Vacuum Atmospheres company, Genesis model). The H_2_O and O_2_ contents in the glovebox are maintained at less than 2 ppm and less than 1 ppm, respectively. All the experiments are conducted inside the inert environment of the glovebox at an average temperature of 20 ± 2 °C unless mentioned otherwise.

A polycrystalline gold working electrode (2 mm diameter disk) has an electrochemically active surface area of 0.033 ± 0.010 cm^2^, calculated by collecting scan-rate-dependent (i.e., 100, 75, 50, 25 mV s^–1^) cyclic voltammetry data of potassium ferricyanide (K_3_[FeCN_6_]) in 0.1 M aqueous KCl. The value obtained for the electroactive surface area is close to the geometric surface area of the electrode (0.031 cm^2^). The calculation of the electroactive surface area of the gold working electrode is shown in SI 2.

The capacitance values are normalized with respect to the effective surface area of the gold working electrode, and all the capacitance measurements are performed in triplicate (*n* = 3). The data are shown as an average capacitance density value with standard deviations.

The working electrode is polished with an aqueous slurry of 1.0 and 0.3 μm MicroPolish II alumina oxide powder (Buehler) on microcloth PSA pads (Buehler), respectively. Between the polishing steps, the surface is sonicated for 5 min in ultrapure water and rinsed with copious amounts of ultrapure water. Immediately after it is polished, the electrode is immersed in a clean beaker of ultrapure water, sealed with parafilm, and stored until use. Immediately before use the surface is removed from the ultrapure water and dried with high-purity nitrogen (N_2_) gas (Praxair high purity 99.998%).

Platinum wires (99.999% metals basis, Alfa Aesar) serve as both counter and reference electrodes. These are cleaned in a hydrogen (H_2_) flame (Praxair UHP H_2_99.999%) prior to being placed into the electrochemical cell. The platinum wire quasi-reference electrode is calibrated with respect to the ferrocene/ferrocenium (Fc/Fc^+^) redox couple at the end of data collection by the addition of a trace amount of ferrocene (Fc, Sigma-Aldrich, ≥98%) to the IL. The average potential of the Fc/Fc^+^ reversible couple derived from direct-current (DC) cyclic voltammetry is scaled to 0.000 V by subtracting the average potential value of the oxidation and reduction peaks of ferrocene from each potential to refer to the potential axis with respect to ferrocene.

A CH Instruments 660D potentiostat (CH Instruments) with shielded potentiostat cables with a capacitance of ∼30 pF per foot is used to perform all the electrochemical measurements. CH Instruments 660D software is used for the data analysis, and from the software, cyclic voltammetry, impedance-potential, and AC Voltammetry techniques are selected to run the cyclic voltammetry, single-frequency impedance, and AC voltammetry experiments, respectively.

The potential region in which these capacitance measurements are performed is determined using DC cyclic voltammetry at a scan rate of 100 mV s^–1^. This helps us to obtain the electrochemical stability window over which no faradaic processes are observed.

The capacitance values are obtained using the single-frequency impedance-potential technique. This method idealizes the system as a simple resistor-capacitor (RC) equivalent circuit model in which the solution resistance (*R*_s_) is in series with a double-layer capacitance (*C*_dl_). The equivalent circuit is shown in SI 3. For such a system, the double-layer capacitance (*C*_dl_) in Farads could be written using eq 2.

Single-frequency capacitance equation.
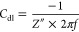
2Here *f* is the applied AC frequency in hertz, and *Z*″ is the imaginary impedance in ohms.^52−57^

AC voltammetry is also a single-frequency impedance technique, and its data are converted into double layer capacitance (*C*_dl_) via eq 3.

AC voltammetry capacitance equation.
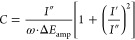
3Here *I*′ and *I*″ are the in- and out-of-phase currents, respectively, ω = 2π*f*, and Δ*E*_amp_ is the perturbation amplitude.^58^ This method also treats the system as a simple RC electrochemical equivalent circuit, which aids the comparison of data to a single-frequency impedance technique.^53^ Both of these single-frequency measurements are performed using a frequency of 10 Hz with an AC voltage of 10 mV and data point collection every 10 mV to maintain the pseudolinearity of the system.^59^SI 4 shows an illustration of the single-frequency data analysis.

Conductivity measurements are performed using the conductivity bench meter SevenCompact S230-Std-Kit (Mettler-Toledo) with an InLab 751 4 mm probe (Mettler-Toledo). Prior to conductivity measurements, the conductivity electrode is immersed into the conditioning solution of 0.5% cleaning reagent and 99.5% deionized water (VWR International, Inc.) overnight (<72 h) to optimize its performance. When ready for use, the electrode is removed from the conditioning solution and is rinsed thoroughly with ultrapure water followed by blot drying. The conductivity meter calibration is performed manually by entering the cell constant value (i.e., 1.000 cm^–1^) default for the InLab 751 4 mm probe. The electrode is cleaned profusely with ultrapure water in between the conductivity measurements. It is important that the probe is dried well before taking conductivity measurements in IL samples. All the conductivity measurements are performed at the two temperatures of 25 and 60 °C. When the conductivity is measured at 25 °C, the auto temperature correction in the meter is enabled. The temperature correction is switched off when the conductivity values are measured at 60 °C.

Viscosity measurements are performed using the DV2T programmable (cone and plate) Brookfield viscometer (Brookfield engineering laboratories), and CPA-40Z is used as the spindle. The temperature is controlled with a precision of 0.1 K using a TC-602 bath thermostat with a Brookfield temperature controller unit attached. Before use, the viscometer is calibrated with a standard oil having a viscosity value of 29.50 cP (mPa·s) at 25 °C. Viscosity measurements of each IL are taken at 25 and 60 °C, unless mentioned otherwise. The instrument is thermally equilibrated at the desired temperature (i.e., at 25 and 60 °C) for 2 min in between each measurement.

All DOSY experiments are conducted on the Bruker Avance III400 MHz spectrometer with automatic tuning and matching BBFO probe equipped with a z-gradient coil. All the measurements are performed at 25 and 60 °C unless mentioned otherwise, and samples are thermally equilibrated at the stated temperature for 15 min before the data collection. All the diffusion measurements are made using the stimulated echo pulse sequence with bipolar gradient pulses.^60,61^ The diffusion delay (Δ) is varied from 20 to 50 ms, and the gradient pulse duration (δ) is varied from 1.5 to 5 ms. Both Δ and δ are optimized to obtain 1–5% residual signal at 95% of the maximum gradient strength. The recycle delay is set to 5 s. Rectangular shapes are used for the gradients, and a linear ramp with 16 increments between 2 and 95% of the maximum gradient strength (*g*_max_ = 100%, 56.0 G/cm) at a current of 10 A is used in all the measurements. The self-diffusion of the residual HDO signal in a pure D_2_O (99.98% D) sample is measured at 25 °C to calibrate the gradient strength. The diffusion coefficients are calculated by integrating the peaks of interest and by a direct curve fitting to the Stejskal-Tanner equation given as eq 4.
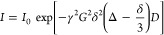
4Here *I* is the observed intensity, and *I*_0_ is the signal intensity in the absence of the gradient. γ is the proton magnetogyric ratio, and *G* is the gradient strength. The *T*_1_/*T*_2_ analysis in Topspin 3.6.1 is used to perform the NMR data processing and curve fittings of all the peaks.

## 3 Results and Discussion

**Figure 2:**
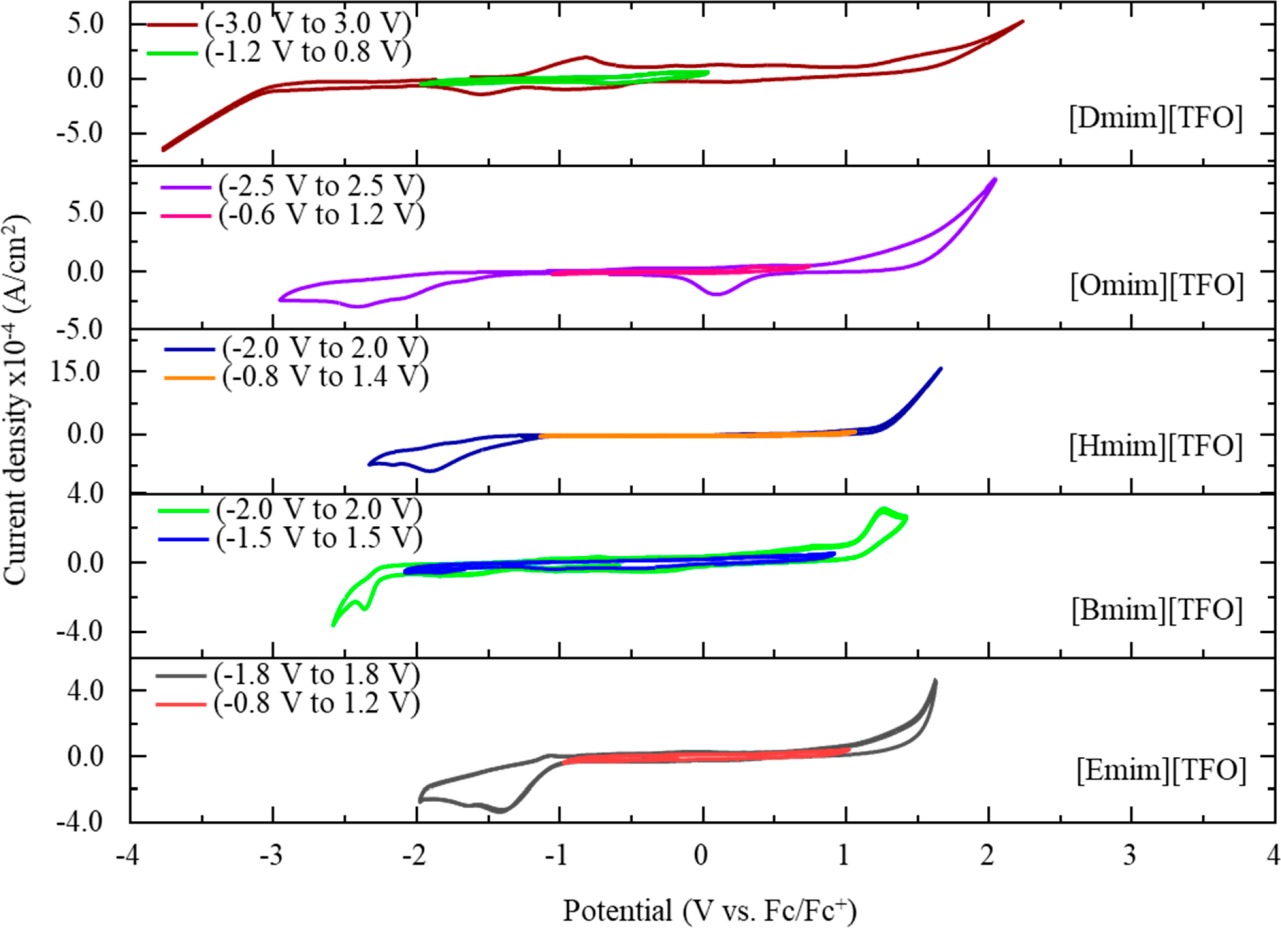
Comparison of potential windows of [Emim] [TFO], [Bmim][TFO], [Hmim] [TFO], [Omim] [TFO], and [Dmim] [TFO] at a polycrystalline gold working electrode. Cyclic voltammograms are obtained at a scan rate of 100 mV s^–1^. The potential axis is given with respect to Fc/Fc^+^.

The aim of this study is to identify the effect of cation chain length on capacitance–potential relationships in ILs. The five ILs examined here share the same anion, triflate (trifluoromethanesulfonate), and the cation differs only in the alkyl chain length, accessing a significant range of bulk viscosity/conductivity values. The stable electrochemical potential windows and double-layer electrochemical potential region for this series of ionic liquids used in our work are given in SI 1, as derived from cyclic voltammetry measurements shown in Figure 2.

### 3.1 DC Voltammograms for IL Systems

Selecting an appropriate electrochemical stability window over which to perform capacitive measurements can significantly impact any study of IL capacitance behavior. The stable voltage window is sensitive to the type of IL, the working electrode employed, and any impurities (e.g., halides and water) that may be present. Consequently, water oxidation, hydrogen evolution, and the formation of metal oxides at the electrode surface may affect the potential window.^62^ Even hydrophobic ILs absorb considerable amounts of water from atmospheric water vapor, and an increase in the water content narrows the electrochemical stability window of ILs at both cathodic and anodic limits.^63^ Therefore, ILs must be dried prior to experiments.^63−67,63,68^ In spite of these precautions, the definition of the electrochemical stability window is not precise. A commonly used diagnostic is to define an arbitrary current density at which the system is no longer “stable” and to assign the potential window not to exceed this current density.^69^ However, even small amounts of current that work to degrade the liquid or electrode can result in impurities or defects that impact the system performance. In our work the stable potential window for each IL examined is shown on the same axis as a wider cyclic voltammogram in Figure 2.

We defined the electrochemical stability window as the rectangular area of the cyclic voltammogram in which the redox currents are minimized. This “double-layer region” should represent the potential window in which the current trace shows minimal or zero oxidation or reduction peaks (no faradaic reactions). The [Omim][TFO] and [Dmim][TFO] showed the largest potential windows spanning over 5.0 and 6.0 V, respectively. The [Hmim][TFO] and [Bmim][TFO] gave potential windows of 4.0 V, and [Emim][TFO] showed a potential window of 3.6 V. These values are similar to those reported in prior literature.^70,71,55,58^ The residual features appearing in the CV of [Dmim][TFO] (Figure 2) at ca. −1.5 and −0.75 V and ∼0.1 V in the CV of [Omim][TFO] with reference to Fc/Fc^+^ could possibly be due to the water impurities present, which cannot be eliminated by drying the ILs under a Schlenk line. We also acknowledge that small levels of impurities could remain from an IL synthesis/metathesis. The determined potential windows for each are compared to other literature published data in SI 1. However, the water content of the ionic liquids in literature sources was not reported.

We note that our observed stable window does not create a clear trend with the cation chain length. Yoshimoto and co-workers have done a similar study to understand the dependence of the potential window on the alkyl chain length of the imidazolium cation, using five imidazolium ILs based on the [TF_2_N]^−^ anion on an Au(111) electrode, and potential windows were defined using a cutoff current density value of 80 μA cm^–2^. They also found that stability, defined as the electrochemical potential window of each IL, was independent of the alkyl chain length in the imidazolium cation.^72^ However, when [Dmim][TFO] and [Emim][TFO] are compared, it is noticeable that the increasing cation chain length of the imidazolium cation significantly increases the cathodic limit to a more negative value (i.e., from −0.8 to −1.2 V) and reduces the anodic limit (from +1.2 to +0.8 V), though there are deviations in the trend for other ILs studied. This observation agrees with the prior literature that the increased cation chain length may have increased the solvophobic interactions, resulting in greater amounts of cohesive energy within the layers formed at the cathode-IL interface.^73,74^ Therefore, on the one hand, more negative potentials are required to reduce the imidazolium cation. On the other hand, longer alkyl chains should create relatively weaker Columbic interactions. Hence, cations with longer neutral tails will experience weaker ion pairing, which will lower the threshold positive potential to oxidize the anion.^73^ Previous electronic structure calculation studies of Margulis et al. on [TF_2_N]^−^ and [NO_3_]^−^ anion-based ILs suggest that the cation is not always reduced first; instead, the localization of the excess electron in ILs is determined by the cation and anion’s highest occupied molecular orbital (HOMO)/lowest unoccupied molecular orbital (LUMO) gaps and, specifically, by their relative LUMO alignments.^75^ Hence, further computational studies on imidazolium triflate ILs are encouraged to aid the design of ILs with varied electronic structures of cations and anions, which is controlled by their chemical nature.

### 3.2 Ionic Liquid Viscosity

**Figure 3:**
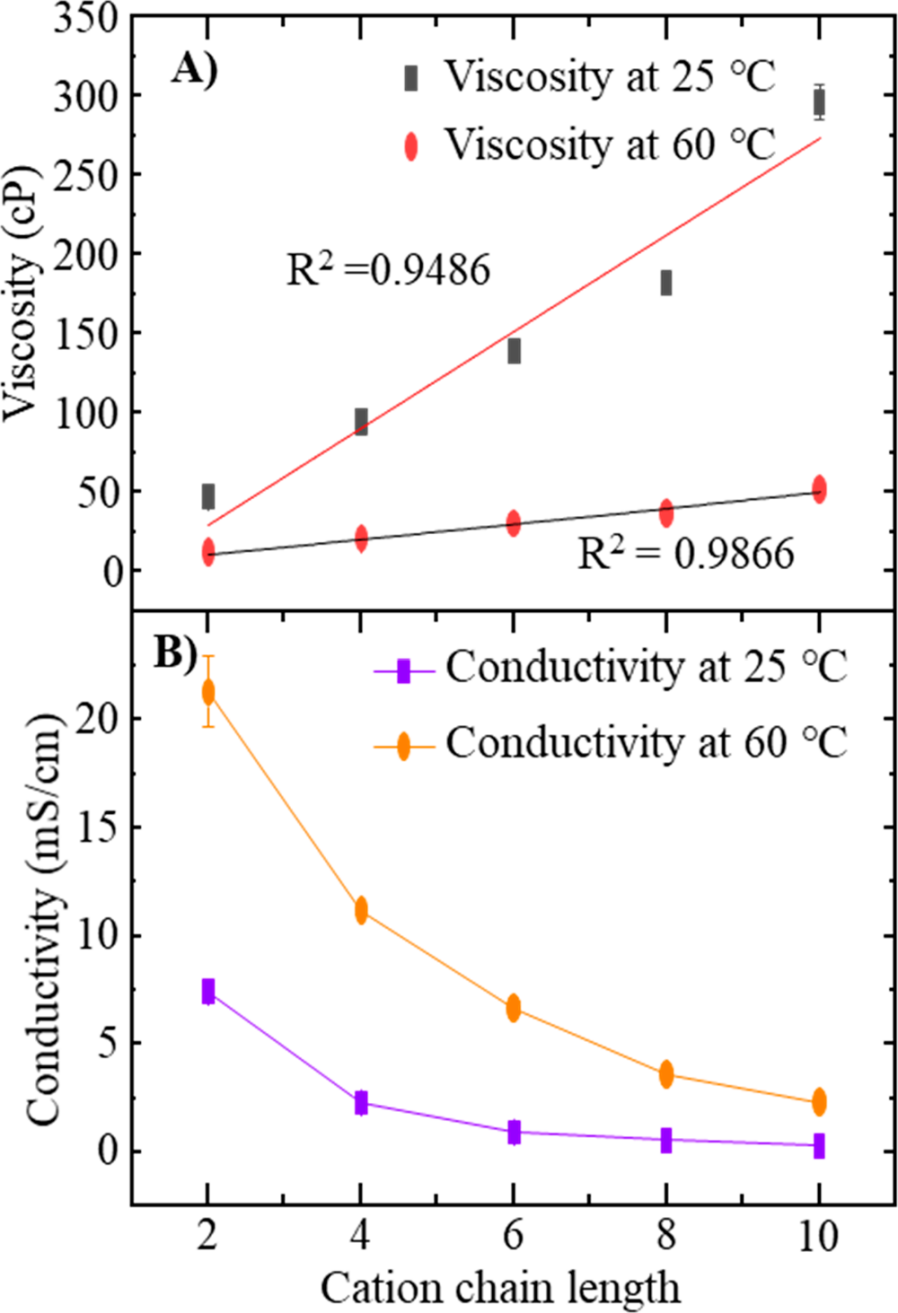
(A) Viscosity data variation with respect to cation alkyl chain length, recorded at 25 and 60 °C. (B) Conductivity data variation with respect to cation chain length at 25 and 60 °C. Values shown here for each chain length are the average value of five independent measurements. Error bars are included, occasionally contained within the data point.

The measured viscosity data at 25 and 60 °C in comparison to previously reported values for each IL are shown in SI 11. The results of the present work are similar to those of previous work.^36,76^Figure 3A shows a viscosity variation with respect to the cation chain length. The obtained viscosity values are the average values from five independent measurements. When the chain length of the imidazolium cation is increased, the viscosity increases as expected. Specifically at 25 °C, the viscosity varies from 47 ± 1 to 296 ± 11 cP, following the order [Emim][TFO] < [Bmim][TFO] < [Hmim][TFO] < [Omim][TFO] < [Dmim][TFO], as previously reported for the same cations paired with [CF_3_SO_3_]^−^,^36^ [NTF_2_]^−^,^77,78^ and [BF_4_]^−^.^79^ At 60 °C, the IL viscosity varies from 12 ± 1 to 52 ± 1 cP. The trend with the alkyl tail length is the same at the elevated temperature. According to Figure 3A, the slope of the line is steeper for 25 °C than for 60 °C, and the viscosity values at 25 °C are at least four units of magnitude higher than the observed viscosity values at 60 °C.

As the imidazolium alkyl tail is lengthened from C2 to C10, we expect Columbic attractions to decrease while van der Waals interactions increase. The interplay of these interactions as well as hydrophobic interactions of the cation tails will dictate the bulk viscosity.^80,81^ Consequently, viscosity increases upon increasing the cation chain length.

### 3.3 Conductivity

Figure 3B shows the variation of conductivity with respect to cation chain length. The conductivity values at 25 °C span the range from 0.31 ± 0.06 to 7.44 ± 0.35 mS/cm and follow the order of [Dmim][TFO] < [Omim][TFO] < [Hmim][TFO] < [Bmim][TFO] < [Emim][TFO]. The estimated standard deviation values for ILs in between different trials differ in the range of 0.01–0.35. Tabluated data for each IL are shown in SI 12 as average values of five independent measurements, and they are similar to those of previously published works.^36,76^ As the ionic conductivity is related to the viscosity, we see that the cation chain length and conductivity are inversely related. Furthermore, the decrease in conductivity upon increasing the chain length can also be described in relation to increasing the steric hindrance with the chain length.

When the temperature is increased to 60 °C, the conductivity increases as viscosity decreases. [Emim][TFO] has the highest conductivity at both temperatures. The conductivity difference at two different temperatures (i.e., 25 and 60 °C) decreases when the cation chain length is increased. For instance, the conductivity difference between 25 and 60 °C for [Emim][TFO] is 14 mS/cm, while that difference is only 2 mS/cm for [Dmim][TFO]. The viscosity difference increases when the cation chain length is increased at the two temperatures, and it would be the most probable reason for this observation. However, it is important to note that viscosity is not the only factor that contributes to the conductivity. Other factors such as ion size, charge delocalization, IL density, aggregation, and correlated ionic motion also play significant roles.^76^ Recent molecular dynamics studies suggest that the viscosity is linked to a nanostructural heterogeneity in which the viscosity is governed by the interplay between intrinsically “stiff” charged networks and “softer”, flexible, and mobile charge-depleted regions.^82^ Hence, it is more likely that the conductivity could be affected by the factors manifested in the bulk viscosity as well as by the molecular origins of viscosity in ionic liquids.

According to Figure 3A, [Omim][TFO] shows the highest variation from the linear trend. On the basis of these data, it is possible that the [Omim][TFO] creates a different nanostructure than the other ILs. However, more experimental evidence could be gathered via computer simulation studies and spectroscopy studies to test this phenomenon.

### 3.4 Ionic Liquids Self-Diffusion

**Figure 4:**
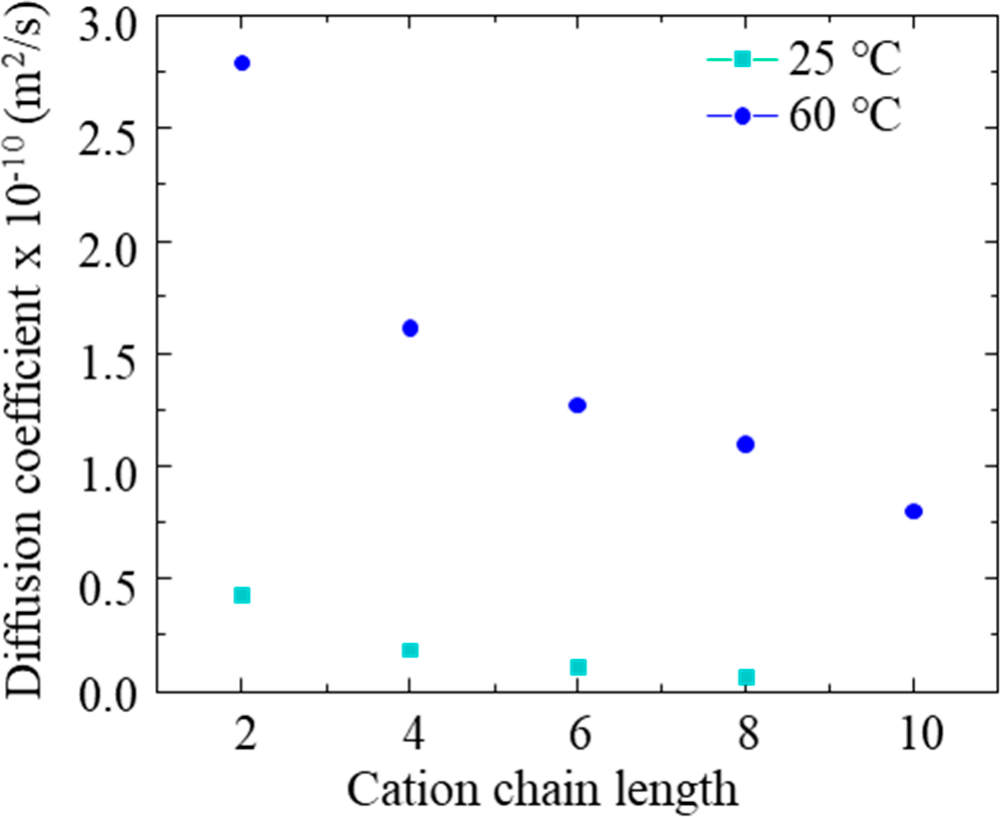
Diffusion coefficient data variation with respect to cation chain length at 25 and 60 °C.

Diffusion-ordered NMR spectroscopy experiments are performed to compute the diffusion coefficients of ILs studied. ^1^H DOSY NMR experiments are performed to calculate the diffusion coefficient of the imidazolium cation, and ^19^F DOSY NMR experiments are performed to calculate the diffusion coefficient of the trifluoromethanesulfonate/triflate anion. Diffusion coefficients provide measures of IL transport properties and are helpful in a structural characterization.^83^ The NMR computed diffusion coefficients of each IL are tabulated in SI 13. The experiments are performed at two different temperatures (i.e., 25 and 60 °C) to study the effect of temperature on the ability of ionic diffusion and potential ion association/dissociation. As per the data shown in Figure 4, the diffusion coefficient for the cation follows the order [Dmim]^+^ < [Omim]^+^ < [Hmim]^+^ < [Bmim]^+^ < [Emim]^+^. The effect of increased alkyl chain length on diffusion is the same as that of the trends we observe for two other transport properties, conductivity and viscosity, namely, that the larger ions move more slowly. The cation diffusion coefficient data of [Emim][TFO] (i.e., 4.3 × 10^–7^ cm^2^/s) and [Bmim][TFO] (i.e., 1.8 × 10^–7^ cm^2^/s) match well with its prior reported diffusion coefficient data (4.1 × 10^–7^ and 1.7 × 10^–7^ cm^2^/s for [Emim]^+^ and [Bmim]^+^ cations, respectively).^36,84^ To our knowledge, the diffusion coefficients of other ILs are measured for the first time in this work.

On the basis of our ^19^F DOSY NMR measurements, the triflate anion diffuses more slowly than the [Emim]^+^ cation (i.e., diffusion coefficients are 2.6 × 10^–7^ cm^2^/s for the triflate anion and 4.3 × 10^–7^ cm^2^/s for the [Emim]^+^ cation). Even though [Emim]^+^ is larger (ionic volume calculated to be ∼116 Å^3^) than the triflate anion (ionic volume calculated to be ∼80 Å^3^),^85^ the boardlike shape of the imidazolium cation could facilitate its faster diffusion relative to triflate.^36^ Hence, it is important to note that size, shape, and intermolecular interactions all affect the ion diffusion.

Ionic self-diffusion coefficients, which characterize microscopic mass transport, are related to the system hydrodynamics (macroscopically obtained viscosity) via the Stokes–Einstein equation, in which the diffusivity is inversely proportional to the fluidity or viscosity. However, the Stokes–Einstein equation is derived from classical hydrodynamics (Stokes’ law) and assumes the diffusing species as a rigid sphere, diluted in an ideal solvent. IL systems contain asymmetrical ions with real ion–ion interactions, and the ions are at high concentrations, which reduces the accuracy of classical models.^86^ Molecular simulation studies of Koddermann et al.^87^ and NMR-based diffusion coefficient experimental studies of Husson et al.^36^ have concluded the nonapplicability of the Stokes–Einstein equation in imidazolium bis(trifluoromethylsulfonyl)imide and imidazolium triflate ILs, respectively. They find that the ion size is not the sole contributor that controls the diffusion. The presence of dynamic heterogeneities and different solvent–solvent interactions also govern the diffusion properties of ILs, irrespective of having similar radii for the cation and anion.^36,87^ To validate the applicability of the Stokes–Einstein equation in ILs here, we calculated the hydrodynamic radius based on the experimental viscosity and diffusion coefficient value of [Bmim]^+^ cation at 25 °C in a pure IL form. The calculated hydrodynamic radius of the [Bmim]^+^ cation at 25 °C is equal to 0.127 nm, which is close to the reported value (0.13 nm)^36^ for the same. However, the van der Waals radius is ca. 0.33 nm,^88^ which raises concerns for the validity of the Stokes–Einstein equation in IL systems.

## 4 Relating Ionic Association, Conductivity, and Viscosity

A limited ionic conductivity is a disadvantage in the use of ILs as electrolytes^89^ and in electrochemical devices^90^ including lithium ion batteries,^91^ supercapacitors,^92−94^ fuel cells,^95^ and photoelectrochemical cells.^96^ Angel and co-workers have described a qualitative approach based on the Walden plot to identify the degree of ion association with respect to KCl, which is fully dissociated.^97,98^ On the basis of the Walden rule, the product of ionic conductivity and viscosity is a constant at a specific temperature.^41^ Most materials (excepting, e.g., LiAlCl_4_ and polyoxometalates),^51,99,100^ fall below the Walden plot’s “KCl line”, as their conductivities are lower than the value predicted by the Walden rule.^101^ In this context, the Walden plot not only indicates the ionic association of ILs with respect to KCl but also it compares the degree of ion correlation of imidazolium triflate ILs with respect to cation chain length.

**Figure 5:**
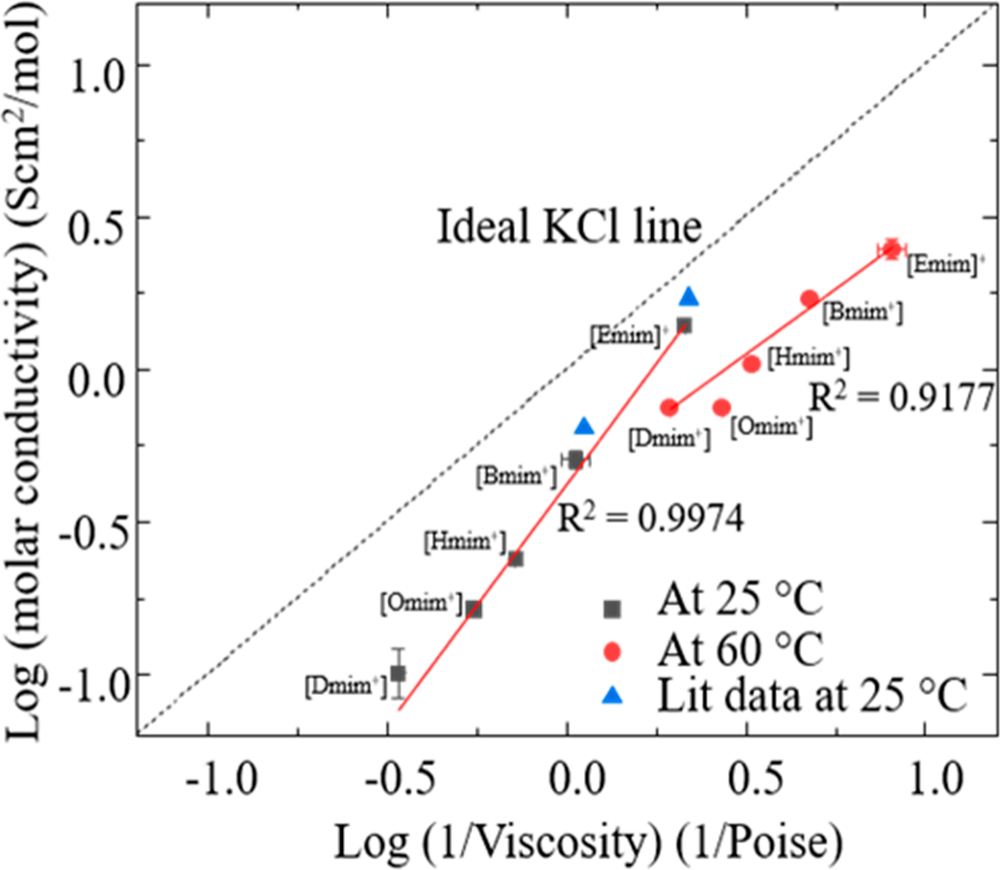
Log–Log plot of molar conductivity vs viscosity (Walden plot) indicates the degree of ionicity of the IL. The plots are drawn using the data collected at 25 and 60 °C and are compared to the “ideal” case of 0.1 M KCl, which exists as dissociated ions. Error propagation is performed for *x* and *y* errors and plotted in the same plot as logarithmic errors.

A Walden plot for the ILs studied here is shown in Figure 5. An error propagation is performed for standard deviation values of individual measurements of the conductivity and viscosity and are shown as error bars in the plot. We note that many of the error bars fall within the size of the data points shown on this log–log plot. Literature values of [Emim]TFO] and [Bmim][TFO] are also plotted in the same Walden plot for comparison, taken from refs (36) and (76). According to this figure, all the ILs studied lie below the ideal KCl line, which indicates a low ionicity or less dissociation with respect to KCl.^101,102^ [Emim][TFO] and [Bmim][TFO] lie closer to the KCl line compared to other three ILs, that is, [Hmim][TFO], [Omim][TFO] and [Dmim][TFO], at 25 °C.

According to the data we obtained at 60 °C, [Emim][TFO] and [Bmim][TFO] seem to have a high degree of cation–anion association, as they lie far away from the ideal KCl line relative to lower temperatures. These results are contradictory with the conductivity, viscosity, and diffusion coefficient data trends obtained for the same. On the one hand, Hussen and co-workers also observed a slight decrease in ionicity when the temperature was increased and suggested that the temperature has no effect on the ionic association.^36^ On the other hand, the [Dmim][TFO] has a high ionicity at 60 °C compared to 25 °C, which is also be reflected from its high conductivity value. The [Omim][TFO] deviates from the above trend at 60 °C showing a much lower ionicity compared to the rest. Harris also observed inconsistencies when applying the Angell-Walden analysis and concluded that this analysis does not convey quantitative information on ionicity other than a qualitative ranking of the conductivity of ILs at a given viscosity and, hence, may be impaired when classifying IL interactions.^103^

## 5 Cation Chain Length Effect of Capacitance–Potential Relationships

Despite the recent widespread use of ILs, information related to an IL-solid interfacial structure is scarce.^104^ Many processes, including energy storage in supercapacitors, occurs via a charge separation at the electrode-IL interface. Therefore, we also report energy-related properties such as interfacial capacitance and energy density. Imidazolium cation-based ILs have been widely used in EDL capacitors, and the identity of the IL’s cation is known to affect the double-layer capacitance.^23,105^

The interfacial capacitance is affected by various factors including the effective permittivity of the IL, topography, and the material of the electrode surface.^29^ Among all the other factors, the alkyl chain length has a great influence in tuning the interfacial structure via viscosity, conductivity, and capacitance changes. As discussed previously in Section 3.3 and Section 3.4, when the alkyl chain length gets larger, the viscosity becomes larger and conductivity gets smaller due to an increasing steric hindrance introduced by the alkyl chain.^24^ With the context of the bulk viscosity and conductivity data reported above, the addition of the electrochemical capacitance versus potential relationships aid in developing an understanding of how the alkyl chain length affects the structure and behaviors of an electrochemical interface when negative and positive potentials are applied. To preserve the overall physicochemical properties determined by a basic ionic structure, we consider five cations in which all share the same cation head but have various neutral chain lengths. We also compare the capacitance obtained from two different single-frequency techniques, namely, single-frequency impedance and AC voltammetry as well as effects of cathodic and anodic scanning directions.

**Figure 6:**
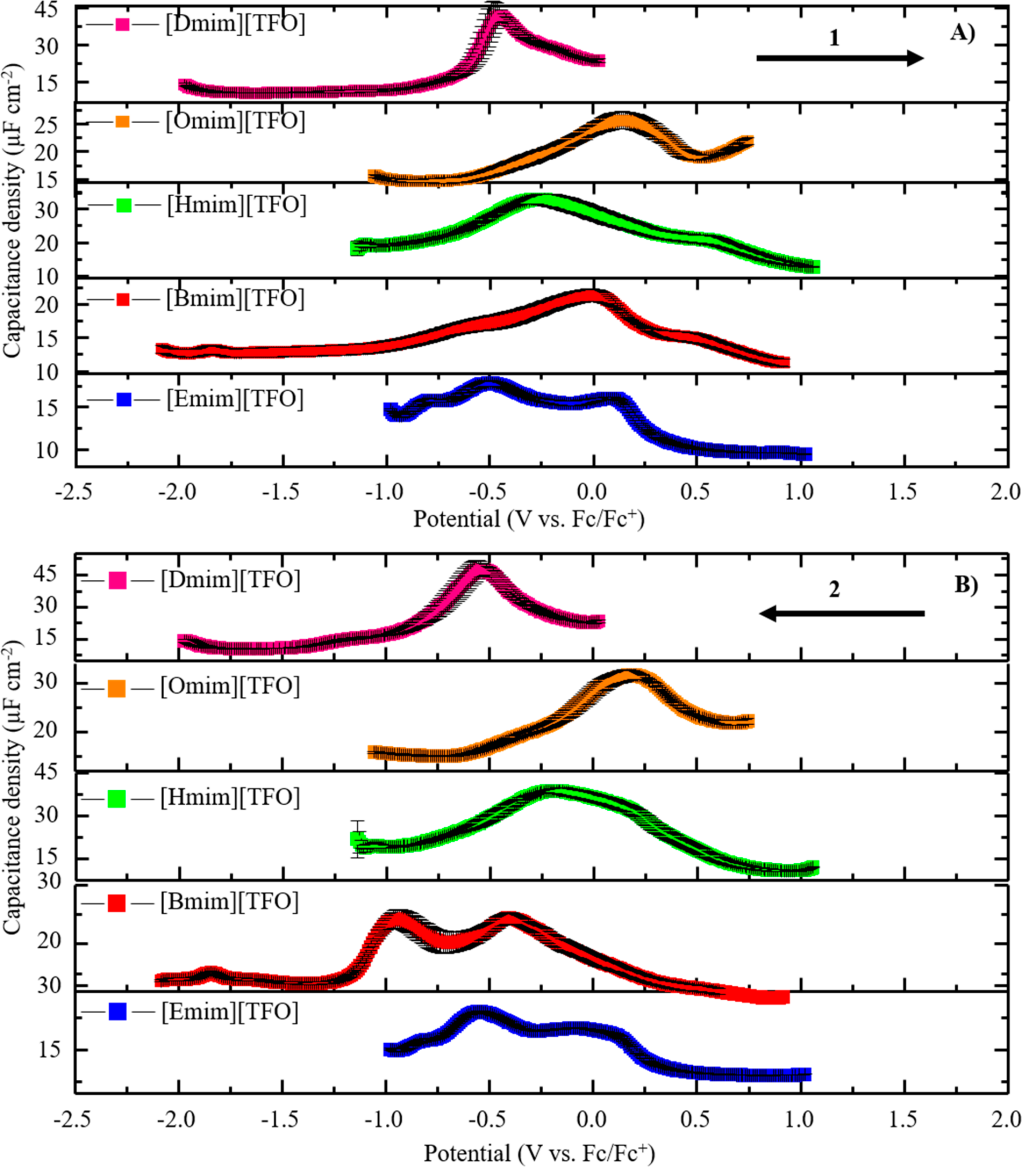
Single-frequency impedance data for five different ILs ([Emim][TFO], [Bmim][TFO], [Hmim][TFO], [Omim][TFO], and [Dmim][TFO]) on a polycrystalline gold electrode obtained in the (A) anodic direction and (B) cathodic direction. The arrow denotes the potential scan direction, and the arrows are numbered to indicate which scan direction is performed first. The anodic direction is performed first followed by the cathodic direction. Error bars represent the standard deviation of three replicate measurements under the same experimental conditions.

Figure 6 shows the capacitance curves in both anodic and cathodic directions, and we compare capacitance of anodic and cathodic scanning directions by taking the maximum capacitance into account. Numerical maximum capacitance values are tabluated in SI 14. Our data show a general trend to increase the maximum capacitance with the increasing chain length of the imidazolium cation, except for [Omim][TFO]. On the basis of the purity testing studies (i.e., Karl Fischer and ^1^H NMR studies) [Omim][TFO] behaves similarly to the other ILs. This claims that the impurities, mainly water, are not the cause for this action. However, in Figure 3, [Omim][TFO] showed the largest deviation from the viscosity linear trend (i.e., by 31.5 viscosity units). On the basis of our experimental observation and literature precedence,^82^ as discussed in Section 3.4, we suspect that the [Omim][TFO] displays a different structural heterogeneity compared to other ILs studied.

The longer alkyl chain of the cation may produce strong solvophobic forces that increase the cohesive forces within layers near the electrode surface. Hence, cations with longer neutral tails will more likely form interfacial structures with an efficient packing. Consequently, the long alkyl chain of the cation could work to reduce the thickness of the EDL structure formed at the electrode, and the thinner electrochemical double layer could contribute to an increasing capacitance and energy density.^106,107^ This phenomenon is supported by Vericat and co-workers’ report based on self-assembled monolayers (SAMs) of thiols on gold surfaces of varying hydrocarbon chain lengths. According to their study, the interactions among backbone hydrocarbon chains involving van der Waals and hydrophobic forces ensure an efficient packing of the monolayer and contribute to stabilize the structures with an increasing chain length.^108^ The results of an early study by Grahame based on the dependence of the integrated capacitance of the mercury/aqueous electrolyte interface on the identity of alkali cations in solution (0.1 M) are also consistent with our interpretation of cation chain length effect on the reduction of the double-layer thickness when the cation size is increased.^109^ In this study, they showed that the Cs^+^ ion resides closer to the interface (compared to Li^+^) due to a partial dehydration of the ion, and the shorter double-layer width accounts for the increase in capacitance.^109,110^

Bhargava et al. highlight the preferability of the [Omim]^+^ cation to retain their orientation with respect to the interface normal for a longer time compared to the [Emim]^+^ cation.^111^ Further, Mao and co-workers’ in situ STM characterizations also demonstrate the strong interaction of the [Omim]^+^ cation with the gold electrode, forming micelle-like structures.^23^ As a result of the [Omim]^+^ cation’s higher retention at the interface, driven by the van der Waals interactions between the neutral counterparts of the cations, they will more likely form more cation layers at the electrode, increasing the EDL thickness and, hence, reducing the capacitance. As the [Dmim]^+^ cation has stronger van der Waals interactions compared to the [Omim]^+^ cation, introduced by the longer neutral tail, it is possible that the [Dmim]^+^ also forms micelle-like structures. However, no prior literature evidence is found for [Dmim]^+^ to form such structures. It could be more likely due to the steric hindrance introduced by the [Dmim]^+^ cation. However, further studies on this phenomenon are warranted, particularly those that would be able to quantify the balance of intermolecular forces leading to possible organized structures. These might include spatial distribution functions of cations and anions using molecular dynamics (MD) simulations,^112^ analyzing the thickness of the double layer using X-ray reflectometry studies^113^ with respect to applied electrode potential, and molecular ion orientation studies using vibrational spectrosocopy.^114^

Beyond the interesting behavior of [Omim]^+^, our results generally show that there is a statistically insignificant difference between the cathodic scanning direction capacitance values and the anodic scan direction capacitance values. For instance, [Bmim][TFO] gives 21.3 ± 0.9 μF/cm^2^ for the anodic direction and 24 ± 1 μF/cm^2^ for the cathodic direction scans. Starting the capacitance measurements at positive applied potentials should induce anions to specifically adsorb and, eventually, be replaced by cation adsorption when the electrochemical potential is moved to more negative values. However, starting at negative applied potentials would make the initial state likely to include an adsorbed imidazolium cation, which could hinder the adsorption of the smaller anion at more positive applied potentials. Consequently, the anodic direction followed by cathodic direction gives slightly lower capacitance values compared to cathodic followed by anodic direction capacitance values. For instance, in the anodic direction, the capacitance value for an anodic followed by cathodic scanning protocol is 17.8 ± 0.3 μF/cm^2^, while for a cathodic followed by anodic scanning protocol, the capacitance value is only 16.9 ± 0.4 μF/cm^2^ for [Emim][TFO], in the same scanning direction (anodic). We suggest this observation results from the π-electronic interaction of the imidazolium ring with the gold working electrode, which forms a compact double layer.^27^ Furthermore, the negative charge is delocalized in the triflate anion due to the electron-withdrawing effect of the three fluorine atoms and resonance effects. Charge delocalization in the cation only occurs across the imidazolium ring, so the cation has a stronger electrostatic adsorption with the gold interface compared to triflate anion.^115^ Because of the imidazolium ion’s ability to rearrange/reorient the charge carrying head-groups,^116^ it is likely to maximize the counterions near the interfacial layer minimizing the potential difference, but there is not a clear trend in “capacitive hysteresis” for our series of *n*-alkylimidazolium cations.

According to coarse-grain MD simulations performed on uncharged surfaces by Wang and co-workers on n-mim^+^/NO_3_^–^ ILs, polar groups form a polar network due to strong electrostatic interactions, while the neutral chains lie parallel to each other supported by van der Waals interactions between the cationic side chains.^117^ When the neutral chain length is increased, ILs could transition from a spatially heterogeneous to a liquid crystalline-like structure due to the competition between the polar groups’ electrostatic interactions and the side chains’ van der Waals interactions.^117,118^ These literature observations support a possibility that, despite a high viscosity and low conductivity, ILs with extended neutral tails can exhibit a high capacitance owing to high charge density at the interface. Ultimately, our data show clearly that, even when scanning from negative to positive applied potentials, capacitance values increase. We assign this to the longer cation chain lengths and hindrance of anion–anion repulsion, which allows a compressed thickness of the double layer.^119^

As shown in Figure 6, [Emim][TFO] shows camel-shaped capacitance curves in both anodic and cathodic scanning directions with maximum capacitance density values of 17.8 ± 0.3 and 23.8 ± 0.2 μF/cm^2^ in anodic and cathodic scanning directions, while [Bmim][TFO] shows a camel-shaped feature in the cathodic direction with a maximum capacitance value of 21.3 ± 0.9 μF/cm^2^ and a bell-shaped feature in anodic scanning direction with a maximum capacitance value of 24 ± 1 μF/cm^2^. Gore et al. observed the same feature shapes for [Bmim][TFO] capacitance–potential curves when performing a single-frequency impedance in anodic and cathodic scan directions.^58^ For all the other ILs studied; [Hmim][TFO], [Omim][TFO], and [Dmim][TFO] show bell-shaped curves in both anodic and cathodic scan directions with maximum capacitance density values of 33 ± 1, 26 ± 2, and 42 ± 4 μF/cm^2^ in the anodic direction and 39 ± 1, 32 ± 2, and 48 ± 3 μF/cm^2^ in the cathodic direction, respectively. Interestingly, the less-viscous [Emim][TFO] exhibits a minimum hysteresis based on the capacitance curve feature position, while the highly viscous [Dmim][TFO] shows a large hysteresis on the same. For instance, [Emim][TFO] exhibits the maximum capacitance approximately at −0.5 V in both anodic and cathodic scanning directions, while [Dmim][TFO] exhibits the maximum capacitance approximately at −0.5 and −0.6 V in anodic and cathodic directions, respectively. The AC voltammetry data (SI 15) show similar patterns in capacitance–potential curve shapes in anodic and cathodic directions. When the cation chain length is increased, the slow dynamics of the cation would have caused more potential-dependent hysteresis in an interfacial restructuring.

According to Monte Carlo simulations, ions having neutral domains such as the alkyl tails in imidazolium cations exhibit camel-shaped capacitance curves when the neutral tails are replaced by charged moieties via rotations and the translation of ions near the interface.^23,120^ The rearrangement of heads and tails leads to the effective compaction of the counterions at the interface, providing the rising branch of the capacitance curve at a moderate electrode polarization (i.e., ca. −0.5 and 0.0 V in anodic and cathodic directions for [Emim][TFO]). However, when the electrode potentials are increased toward extreme positive or negative potentials, the double layer swells when cations and anions tend to form more ion layers, and the capacitance subsequently falls in magnitude.^120^ According to our results (Figure 6 and SI 15), the capacitance curve shape is transitioning from a camel shape to a bell shape as a function of the alkyl chain length, and imidazolium cations with alkyl tails longer than C6 form only bell-shaped capacitive curves. According to Fedorov and Kornyshev, even though only cations have neutral moieties, the compaction of ions and swelling of the double layer occur at both cathodic and anodic polarizations of the electrode at different applied potentials, which results in the camel-shaped feature.^121^ However, as we increase the neutral chain length, the strong van der Waals interactions impede the formation of cation–anion layering, resulting in the transition from a camel- to bell-shaped capacitive feature. This observation further strengthens our argument of van der Waals interactions between neutral chains competing with, and even overcoming, the Columbic interactions between cations and anions preventing the formation of more ion layers near the interface.

When collecting the capacitance data starting from the cathodic direction followed by the anodic direction, we observe similar trends with respect to the cation chain length and scan direction; the capacitance increases when the chain length is increased, and the cathodic direction capacitance is relatively higher than the capacitance of the anodic direction. These observations can be interpreted in the same way as above. Additional data, showing effects of the acquisition technique and direction of the applied electrochemical potential, are shown in SI 16 and SI 17.

### 5.1 Implications for EDL Energy Storage

Overall, our results demonstrate how the cation alkyl tail length affects the properties of the EDL, specifically, the capacitance versus applied potential. In order to increase the energy density of electrochemical double layer capacitors, both the capacitance and electrochemical stability window (the potential of the double-layer region) of the IL must be considered. While capacitance values rise with longer alkyl chain lengths, as discussed previously in Section 3.1, the potential window does not increase as we increase the chain length of the cation. Hence, we do not observe large changes in the energy density of these ILs (Table 1).

**Table 1:** Tabulated Energy Density (Given in Units of J/cm^2^) for the IL Systems Studied Here, Listed for Measurement Technique (SFI = Single-Frequency Impedance and ACV = Alternating Current Voltammetry) and Direction of the Applied Potential Sweep^a^

	SFI energy density × 10^–6^ (J/cm^2^)				ACV energy density × 10^–6^ (J/cm^2^)			
	anodic to cathodic		cathodic to anodic		anodic to cathodic		cathodic to anodic	
ionic liquid	anodic	cathodic	cathodic	anodic	anodic	cathodic	cathodic	anodic
[Emim][TFO]	36 ± 2	48 ± 1	45 ± 1	34 ± 2	23 ± 8	31 ± 3	29 ± 9	22 ± 1
[Bmim][TFO]	96 ± 4	110 ± 4	105 ± 1	92 ± 2	63 ± 3	86 ± 5	80 ± 4	66 ± 11
[Hmim][TFO]	80 ± 4	94 ± 2	122 ± 13	108 ± 13	51 ± 7	61 ± 4	80 ± 9	78 ± 8
[Omim][TFO]	42 ± 6	51 ± 3	45 ± 1	37 ± 1	26 ± 13	35 ± 2	30 ± 1	25 ± 1
[Dmim][TFO]	84 ± 9	95 ± 7	77 ± 6	59 ± 7	56 ± 4	67 ± 5	56 ± 13	41 ± 10

a Energy density (*E*) is calculated using *E* = (*CV*^2^)/2, where *C* is the maximum capacitance, and *V* is the double-layer region potential window. Error propagation is performed only for the maximum capacitance value as an average of *n* ≥ 3 independent measurements. Calculated uncertainties are shown with the energy density values.

To facilitate a comparison of these values with existing reports we provide these data in units of joules per liter (J/L) in SI 18. Here, we assume the maximum effective Debye length reported for ionic liquids systems (10 nm) as the limiting dimension of a cylinder that contains the active volume of the ionic liquid. The calculation of the true volume of the IL in contact with the gold working electrode is included in detail in SI 18. According to our results, the maximum energy density is achieved by the [Hmim][TFO] IL, and it is calculated to be ∼3670 kJ/L (∼1 kWh/L). While evaluations based on eq 1 are known to overstate the performance of ELDCs, the energy value of [Hmim][TFO] IL is significantly larger than that reported for EDLCs based on molecular solvents such as propylene carbonate or acetonitrile (i.e., 18–28.8 J/L)^122^ but similar to other ionic liquid devices.^123^ Also, the nonflammability and high thermal stability of imidazolium triflate ILs over propylene carbonate/acetonitrile are added advantages allowing a safer deployment of ILs in capacitive energy storage devices.^124,125^

## 6 Conclusion

In this work, three transport properties, namely, viscosity, electrical conductivity, and diffusion coefficient, are experimentally determined at 25 and 60 °C in five imidazolium triflate ionic liquids with different lengths of the imidazolium cation’s alkyl tail. Higher bulk values of viscosity, lower electrical conductivities, and lower diffusion coefficients are measured when increasing the alkyl chain length of the imidazolium cation. Higher conductivities and lower viscosities are observed at 60 °C relative to 25 °C due to reduced cation–anion Columbic interactions, which yield high diffusivities. Walden plots of these data show that the ionicity of the ILs tested here are all below the ideal KCl line, regardless of the temperature or alkyl chain length. In order to relate the observed trends for the bulk properties with the energy-related properties, we also analyze capacitance–potential relationships qualitatively and quantitatively via capacitance measurements using single-frequency and AC voltammetry, in both anodic and cathodic scan directions. Despite the higher bulk viscosities, lower electrical conductivities, and lower diffusion coefficients, the capacitance values generally increase as we increase the cation’s alkyl chain length. We rationalize this as being a consequence of the nonpolar alkyl tail that could weaken the Columbic interactions between ion pairs. This could prevent the formation of multiple layers at polarized electrodes, thinning the double layer, and increasing total capacitance. Hence, it is important to note that the capacitance is most possibly governed by the interfacial properties and not by their bulk properties. [Omim][TFO] deviates from the observed trend, driven by the high retention ability of the [Omim]^+^ cation at the interface. More ion layers would form, leading to more thickness in the double layer, reducing the capacitance. Furthermore, the capacitance–potential curve shape transitions from a camel to bell shape as we increase the alkyl chain length, which is an interesting finding of this work. As both the capacitance and electrochemical stability window of the IL of interest affect the energy density of EDLCs, we do not observe a clear trend in energy density with respect to the cation chain length.

## Supporting Information Available

Details on the calculation of electroactive surface area of the working electrode, potential windows and water contents of each IL, equivalent circuit diagram, ^1^H NMR data of ILs, experimental viscosity, conductivity, and diffusion coefficient values, maximum capacitance values obtained for each IL, calculated energy values for each IL, capacitance–potential profiles obtained from single frequency impedance data and AC voltammetry data, illustration of single frequency data analysis (PDF)

## References

[ref1] Gielen D.; Boshell F.; Saygin D.; Bazilian M. D.; Wagner N.; Gorini R. The role of renewable energy in the global energy transformation. Energy Strategy Reviews 2019, 24, 38–50. 10.1016/j.esr.2019.01.006.

[ref2] Khan K.; Tareen A. K.; Aslam M.; Mahmood A.; Khan Q.; Zhang Y.; Ouyang Z.; Guo Z.; Zhang H. Going green with batteries and supercapacitor: Two dimensional materials and their nanocomposites based energy storage applications. Prog. Solid State Chem. 2020, 58, 100254 10.1016/j.progsolidstchem.2019.100254.

[ref3] Zhang S.; Pan N. Supercapacitors Performance Evaluation. Adv. Energy Mater. 2015, 5 (6), 1401401 10.1002/aenm.201401401.

[ref4] Galiński M.; Lewandowski A.; Stępniak I. Ionic liquids as electrolytes. Electrochim. Acta 2006, 51 (26), 5567–5580. 10.1016/j.electacta.2006.03.016.

[ref5] Liu K.; Lian C.; Henderson D.; Wu J. Impurity effects on ionic-liquid-based supercapacitors. Mol. Phys. 2017, 115 (4), 454–464. 10.1080/00268976.2016.1271154.

[ref6] Kim B. K.; Sy S.; Yu A.; Zhang J. Electrochemical Supercapacitors for Energy Storage and Conversion. Handbook of Clean Energy Systems 2015, 1–25. 10.1002/9781118991978.hces112.

[ref8] Lu P.; Dai Q.; Wu L.; Liu X. Structure and Capacitance of Electrical Double Layers at the Graphene-Ionic Liquid Interface. Appl. Sci. 2017, 7 (9), 939 10.3390/app7090939.

[ref9] Zhang L. L.; Zhao X. S. Carbon-based materials as supercapacitor electrodes. Chem. Soc. Rev. 2009, 38 (9), 2520–2531. 10.1039/b813846j. 19690733

[ref10] Cho J.; Shin W.-K.; Kim D.-W.; Kim Y. R.; Lee B. J.; Kim S.-G. Electrochemical Characterization of Electric Double Layer Capacitors Assembled with Pyrrolidinium-Based Ionic Liquid Electrolytes. J. Electrochem. Sci. Technol. 2016, 7 (3), 199–205. 10.5229/JECST.2016.7.3.199.

[ref11] Xu B.; Wu F.; Chen R.; Cao G.; Chen S.; Wang G.; Yang Y. Room temperature molten salt as electrolyte for carbon nanotube-based electric double layer capacitors. J. Power Sources 2006, 158 (1), 773–778. 10.1016/j.jpowsour.2005.08.043.

[ref12] Holze R. Electrodeposition from ionic liquids. F. Endres, A. P. Abbott, and D. R. MacFarlane (Eds). WILEY-VCH, Weinheim, 2008. J. Solid State Electrochem. 2009, 13, 1633–1634. 10.1007/s10008-009-0821-6.

[ref13] Weingarth D.; Noh H.; Foelske-Schmitz A.; Wokaun A.; Kötz R. A reliable determination method of stability limits for electrochemical double layer capacitors. Electrochim. Acta 2013, 103, 119–124. 10.1016/j.electacta.2013.04.057.

[ref14] Drüschler M.; Huber B.; Passerini S.; Roling B. Hysteresis Effects in the Potential-Dependent Double Layer Capacitance of Room Temperature Ionic Liquids at a Polycrystalline Platinum Interface. J. Phys. Chem. C 2010, 114 (8), 3614–3617. 10.1021/jp911513k.

[ref15] Kudlak B.; Owczarek K.; Namiesnik J. Selected issues related to the toxicity of ionic liquids and deep eutectic solvents--a review. Environ. Sci. Pollut. Res. 2015, 22 (16), 11975–92. 10.1007/s11356-015-4794-y. 26040266

[ref16] Aschenbrenner O.; Supasitmongkol S.; Taylor M.; Styring P. Measurement of vapour pressures of ionic liquids and other low vapour pressure solvents. Green Chem. 2009, 11 (8), 1217–1221. 10.1039/b904407h.

[ref17] Burke A. R&D considerations for the performance and application of electrochemical capacitors. Electrochim. Acta 2007, 53 (3), 1083–1091. 10.1016/j.electacta.2007.01.011.

[ref18] Cheng Z.; Yida D.; Wenbin H.; Daoming S.; Xiaopeng H.; Jinli Q.; Jiujun Z. Compatibility of Electrolytes with Inactive Components of Electrochemical Supercapacitors. In Electrolytes for Electrochemical Supercapacitors*;* CRC Press, 2016.

[ref19] Earle M.; Wasserscheid P.; Schulz P.; Olivier-Bourbigou H.; Favre F.; Vaultier M.; Kirschning A.; Singh V.; Riisager A.; Fehrmann R.; Kuhlmann S. Organic Synthesis. Ionic Liquids in Synthesis 2007, 265–568. 10.1002/9783527621194.ch5.

[ref20] Gallegos A.; Lian C.; Dyatkin B.; Wu J. Side-chain effects on the capacitive behaviour of ionic liquids in microporous electrodes. Mol. Phys. 2019, 117 (23–24), 3603–3613. 10.1080/00268976.2019.1650210.

[ref21] Wallauer J.; Drüschler M.; Huber B.; Roling B. The Differential Capacitance of Ionic Liquid/Metal Electrode Interfaces - A Critical Comparison of Experimental Results with Theoretical Predictions. Z. Naturforsch., B: J. Chem. Sci. 2013, 68, 1143 10.5560/znb.2013-3153.

[ref22] Ignat’ev N. V.; Barthen P.; Kucheryna A.; Willner H.; Sartori P. A convenient synthesis of triflate anion ionic liquids and their properties. Molecules 2012, 17 (5), 5319–38. 10.3390/molecules17055319. 22565482 PMC6268271

[ref23] Su Y.; Yan J.; Li M.; Zhang M.; Mao B. Electric Double Layer of Au(100)/Imidazolium-Based Ionic Liquids Interface: Effect of Cation Size. J. Phys. Chem. C 2013, 117 (1), 205–212. 10.1021/jp3079919.

[ref24] Vatamanu J.; Borodin O.; Bedrov D.; Smith G. D. Molecular Dynamics Simulation Study of the Interfacial Structure and Differential Capacitance of Alkylimidazolium Bis(trifluoromethanesulfonyl)imide [Cnmim][TFSI] Ionic Liquids at Graphite Electrodes. J. Phys. Chem. C 2012, 116 (14), 7940–7951. 10.1021/jp301399b.

[ref25] Yang J.; Lian C.; Liu H. Chain length matters: Structural transition and capacitance of room temperature ionic liquids in nanoporous electrodes. Chem. Eng. Sci. 2020, 227, 115927 10.1016/j.ces.2020.115927.

[ref26] Lockett V.; Horne M.; Sedev R.; Rodopoulos T.; Ralston J. Differential capacitance of the double layer at the electrode/ionic liquids interface. Phys. Chem. Chem. Phys. 2010, 12 (39), 12499–12512. 10.1039/c0cp00170h. 20721389

[ref27] Alam M. T.; Islam M. M.; Okajima T.; Ohsaka T. Capacitance Measurements in a Series of Room-Temperature Ionic Liquids at Glassy Carbon and Gold Electrode Interfaces. J. Phys. Chem. C 2008, 112 (42), 16600–16608. 10.1021/jp804620m.

[ref28] Wallauer J.; Drüschler M.; Huber B.; Roling B. The Differential Capacitance of Ionic Liquid/Metal Electrode Interfaces - A Critical Comparison of Experimental Results with Theoretical Predictions. Z. Naturforsch., B: J. Chem. Sci. 2013, 68 (10), 1143–1153. 10.5560/znb.2013-3153.

[ref29] Jo S.; Park S.-W.; Shim Y.; Jung Y. Effects of Alkyl Chain Length on Interfacial Structure and Differential Capacitance in Graphene Supercapacitors: A Molecular Dynamics Simulation Study. Electrochim. Acta 2017, 247, 634–645. 10.1016/j.electacta.2017.06.169.

[ref30] Ionic Liquid Mixtures As Electrolytes for Electrochemical Capacitors. ECS Meeting Abstracts 2015. 10.1149/MA2015-02/9/589

[ref31] Shahzad S.; Shah A.; Kowsari E.; Iftikhar F. J.; Nawab A.; Piro B.; Akhter M. S.; Rana U. A.; Zou Y. Ionic Liquids as Environmentally Benign Electrolytes for High-Performance Supercapacitors. Global Challenges 2019, 3 (1), 1800023 10.1002/gch2.201800023. 31565352 PMC6383960

[ref32] Cussler E. L. Diffusion, mass transfer in fluid systems/E.L. Cussler*;* Cambridge: Cambridge, UK, 1984.

[ref33] Sequeira M. C. M.; Avelino H. M. N. T.; Caetano F. J. P.; Fareleira J. M. N. A. Viscosity measurements of 1-ethyl-3-methylimidazolium trifluoromethanesulfonate (EMIM OTf) at high pressures using the vibrating wire technique. Fluid Phase Equilib. 2020, 505, 112354 10.1016/j.fluid.2019.112354.

[ref34] Anwar N.; Riyazuddeen Excess Molar Volumes, Excess Molar Isentropic Compressibilities, Viscosity Deviations, and Activation Parameters for 1-Ethyl-3-methyl-imidazolium Trifluoro-methanesulfonate + Dimethyl Sulfoxide and/or Acetonitrile at T = 298.15 to 323.15 K and P = 0.1 MPa. J. Chem. Eng. Data 2018, 63 (2), 269–289. 10.1021/acs.jced.7b00429.

[ref35] Anwar N.; Riyazuddeen; Urooj F. Effect of co-solvent and temperature on interactions in 1-ethyl-3-methylimidazolium trifluoromethanesulfonate+ethylene glycol or/and N, N-dimethylformamide, and ethylene glycol+N, N-dimethylformamide mixtures: Measurement of thermophysical properties. J. Mol. Liq. 2018, 265, 121–134. 10.1016/j.molliq.2018.05.098.

[ref36] Mbondo Tsamba B. E.; Sarraute S.; Traïkia M.; Husson P. Transport Properties and Ionic Association in Pure Imidazolium-Based Ionic Liquids as a Function of Temperature. J. Chem. Eng. Data 2014, 59 (6), 1747–1754. 10.1021/je400841s.

[ref37] Hooper J. B.; Borodin O. Molecular dynamics simulations of N, N, N, N-tetramethylammonium dicyanamide plastic crystal and liquid using a polarizable force field. Phys. Chem. Chem. Phys. 2010, 12 (18), 4635–43. 10.1039/b925946e. 20428543

[ref38] Tsuzuki S. Factors controlling the diffusion of ions in ionic liquids. ChemPhysChem 2012, 13 (7), 1664–70. 10.1002/cphc.201100870. 22511561

[ref39] Zech O.; Stoppa A.; Buchner R.; Kunz W. The Conductivity of Imidazolium-Based Ionic Liquids from (248 to 468) K. B. Variation of the Anion. J. Chem. Eng. Data 2010, 55 (5), 1774–1778. 10.1021/je900793r.

[ref40] Vila J.; Ginés P.; Rilo E.; Cabeza O.; Varela L. M. Great increase of the electrical conductivity of ionic liquids in aqueous solutions. Fluid Phase Equilib. 2006, 247 (1), 32–39. 10.1016/j.fluid.2006.05.028.

[ref41] MacFarlane D. R.; Forsyth M.; Izgorodina E. I.; Abbott A. P.; Annat G.; Fraser K. On the concept of ionicity in ionic liquids. Phys. Chem. Chem. Phys. 2009, 11 (25), 4962–4967. 10.1039/b900201d. 19562126

[ref42] Holbrey J. D.; Rogers R. D.; Mantz R. A.; Trulove P. C.; Cocalia V. A.; Visser A. E.; Anderson J. L.; Anthony J. L.; Brennecke J. F.; Maginn E. J.; Welton T.; Mantz R. A. Physicochemical Properties. Ionic Liquids in Synthesis 2007, 57–174. 10.1002/9783527621194.ch3.

[ref43] Kunze M.; Jeong S.; Appetecchi G. B.; Schönhoff M.; Winter M.; Passerini S. Mixtures of ionic liquids for low temperature electrolytes. Electrochim. Acta 2012, 82, 69–74. 10.1016/j.electacta.2012.02.035.

[ref44] Kurig H.; Vestli M.; Tõnurist K.; Jänes A.; Lust E. Influence of Room Temperature Ionic Liquid Anion Chemical Composition and Electrical Charge Delocalization on the Supercapacitor Properties. J. Electrochem. Soc. 2012, 159, A944 10.1149/2.022207jes.

[ref45] Lei Z.; Liu Z.; Wang H.; Sun X.; Lu L.; Zhao X. S. A high-energy-density supercapacitor with graphene-CMK-5 as the electrode and ionic liquid as the electrolyte. J. Mater. Chem. A 2013, 1 (6), 2313–2321. 10.1039/c2ta01040b.

[ref46] de Souza R. F.; Padilha J. C.; Gonçalves R. S.; Dupont J. Room temperature dialkylimidazolium ionic liquid-based fuel cells. Electrochem. Commun. 2003, 5 (8), 728–731. 10.1016/S1388-2481(03)00173-5.

[ref47] Kim G. T.; Jeong S. S.; Xue M. Z.; Balducci A.; Winter M.; Passerini S.; Alessandrini F.; Appetecchi G. B. Development of ionic liquid-based lithium battery prototypes. J. Power Sources 2012, 199, 239–246. 10.1016/j.jpowsour.2011.10.036.

[ref48] Garcia B.; Lavallée S.; Perron G.; Michot C.; Armand M. Room temperature molten salts as lithium battery electrolyte. Electrochim. Acta 2004, 49 (26), 4583–4588. 10.1016/j.electacta.2004.04.041.

[ref49] Sakaebe H.; Matsumoto H. N-Methyl-N-propylpiperidinium bis(trifluoromethanesulfonyl)imide (PP13-TFSI) - novel electrolyte base for Li battery. Electrochem. Commun. 2003, 5 (7), 594–598. 10.1016/S1388-2481(03)00137-1.

[ref50] Nakamoto H.; Suzuki Y.; Shiotsuki T.; Mizuno F.; Higashi S.; Takechi K.; Asaoka T.; Nishikoori H.; Iba H. Ether-functionalized ionic liquid electrolytes for lithium-air batteries. J. Power Sources 2013, 243, 19–23. 10.1016/j.jpowsour.2013.05.147.

[ref51] Angell C. A.; Byrne N.; Belieres J.-P. Parallel Developments in Aprotic and Protic Ionic Liquids: Physical Chemistry and Applications. Acc. Chem. Res. 2007, 40 (11), 1228–1236. 10.1021/ar7001842. 17979250

[ref52] Copper C. L.; Whitaker K. W. Capillary Electrophoresis: Part II. Applications. J. Chem. Educ. 1998, 75 (3), 347 10.1021/ed075p347.

[ref53] Lucio A. J.; Shaw S. K. Effects and controls of capacitive hysteresis in ionic liquid electrochemical measurements. Analyst 2018, 143 (20), 4887–4900. 10.1039/C8AN01085D. 30183031

[ref54] Nanjundiah C.; McDevitt S.; Koch V. Differential capacitance measurements in solvent-free ionic liquids at Hg and C interfaces. J. Electrochem. Soc. 1997, 144 (10), 3392–3397. 10.1149/1.1838024.

[ref55] Small L.; Wheeler D. Influence of Analysis Method on the Experimentally Observed Capacitance at the Gold-Ionic Liquid Interface. J. Electrochem. Soc. 2014, 161 (4), H260–H263. 10.1149/2.094404jes.

[ref56] Alam M. T.; Mominul Islam M.; Okajima T.; Ohsaka T. Measurements of differential capacitance in room temperature ionic liquid at mercury, glassy carbon and gold electrode interfaces. Electrochem. Commun. 2007, 9 (9), 2370–2374. 10.1016/j.elecom.2007.07.009.

[ref57] Alam M. T.; Masud J.; Islam M. M.; Okajima T.; Ohsaka T. Differential Capacitance at Au(111) in 1-Alkyl-3-methylimidazolium Tetrafluoroborate Based Room-Temperature Ionic Liquids. J. Phys. Chem. C 2011, 115 (40), 19797–19804. 10.1021/jp205800x.

[ref58] Gore T. R.; Bond T.; Zhang W.; Scott R. W. J.; Burgess I. J. Hysteresis in the measurement of double-layer capacitance at the gold-ionic liquid interface. Electrochem. Commun. 2010, 12 (10), 1340–1343. 10.1016/j.elecom.2010.07.015.

[ref59] Giner-Sanz J. J.; Ortega E. M.; Pérez-Herranz V. Optimization of the Perturbation Amplitude for EIS Measurements Using a Total Harmonic Distortion Based Method. J. Electrochem. Soc. 2018, 165 (10), E488–E497. 10.1149/2.1021810jes.

[ref60] Peroza C. A.; Chen F.; Wurster D. E.; Velupillai S. M. Solubilization of organics I: (1) H NMR chemical shift perturbations, diffusometry, and NOESY indicate biphenyls internalize in micelles formed by cetyltrimethylammonium bromide. Magn. Reson. Chem. 2019, 57 (12), 1097–1106. 10.1002/mrc.4891. 31090226

[ref61] Lehman S. E.; Tataurova Y.; Mueller P. S.; Mariappan S. V. S.; Larsen S. C. Ligand Characterization of Covalently Functionalized Mesoporous Silica Nanoparticles: An NMR Toolbox Approach. J. Phys. Chem. C 2014, 118 (51), 29943–29951. 10.1021/jp5099156.

[ref62] Droessler J. E.; Czerwinski K. R.; Hatchett D. W. Electrochemical Measurement of Gold Oxide Reduction and Methods for Acid Neutralization and Minimization of Water in Wet Ionic Liquid. Electroanalysis 2014, 26 (12), 2631–2638. 10.1002/elan.201400450.

[ref63] O’Mahony A. M.; Silvester D. S.; Aldous L.; Hardacre C.; Compton R. G. Effect of Water on the Electrochemical Window and Potential Limits of Room-Temperature Ionic Liquids. J. Chem. Eng. Data 2008, 53 (12), 2884–2891. 10.1021/je800678e.

[ref64] Klein J. M.; Panichi E.; Gurkan B. Potential dependent capacitance of [EMIM][TFSI], [N1114][TFSI] and [PYR13][TFSI] ionic liquids on glassy carbon. Phys. Chem. Chem. Phys. 2019, 21, 3712 10.1039/C8CP04631J. 30334051

[ref65] Cui T.; Lahiri A.; Carstens T.; Borisenko N.; Pulletikurthi G.; Kuhl C.; Endres F. Influence of Water on the Electrified Ionic Liquid/Solid Interface: A Direct Observation of the Transition from a Multilayered Structure to a Double-Layer Structure. J. Phys. Chem. C 2016, 120 (17), 9341–9349. 10.1021/acs.jpcc.6b02549.

[ref66] Silvester D. S.; Compton R. G. Electrochemistry in Room Temperature Ionic Liquids: A Review and Some Possible Applications. Z. Phys. Chem. 2006, 220, 1247 10.1524/zpch.2006.220.10.1247.

[ref67] Zhang J.; Bond A. M. Practical considerations associated with voltammetric studies in room temperature ionic liquids. Analyst 2005, 130 (8), 1132–1147. 10.1039/b504721h. 16021212

[ref68] Schröder U.; Wadhawan J. D.; Compton R. G.; Marken F.; Suarez P. A. Z.; Consorti C. S.; de Souza R. F.; Dupont J. Water-induced accelerated ion diffusion: voltammetric studies in 1-methyl-3-[2,6-(S)-dimethylocten-2-yl]imidazolium tetrafluoroborate, 1-butyl-3-methylimidazolium tetrafluoroborate and hexafluorophosphate ionic liquids. New J. Chem. 2000, 24 (12), 1009–1015. 10.1039/b007172m.

[ref69] Mousavi M. P. S.; Dittmer A. J.; Wilson B. E.; Hu J.; Stein A.; Bühlmann P. Unbiased Quantification of the Electrochemical Stability Limits of Electrolytes and Ionic Liquids. J. Electrochem. Soc. 2015, 162 (12), A2250–A2258. 10.1149/2.0271512jes.

[ref70] Ohno H. Electrochemical aspects of ionic liquids/edited by Hiroyuki Ohno*;* Wiley: Hoboken, NJ, 2011.

[ref71] Meng T.; Young K.-H.; Wong D.; Nei J. Ionic Liquid-Based Non-Aqueous Electrolytes for Nickel/Metal Hydride Batteries. Batteries 2017, 3 (1), 4 10.3390/batteries3010004.

[ref72] Yoshimoto S.; Taguchi R.; Tsuji R.; Ueda H.; Nishiyama K. Dependence on the crystallographic orientation of Au for the potential window of the electrical double-layer region in imidazolium-based ionic liquids. Electrochem. Commun. 2012, 20, 26–28. 10.1016/j.elecom.2012.03.049.

[ref73] Li H.; Endres F.; Atkin R. Effect of alkyl chain length and anion species on the interfacial nanostructure of ionic liquids at the Au(111)ionic liquid interface as a function of potential. Phys. Chem. Chem. Phys. 2013, 15 (35), 14624–14633. 10.1039/c3cp52421c. 23873270

[ref74] Sun L.; Morales-Collazo O.; Xia H.; Brennecke J. F. Effect of Structure on Transport Properties (Viscosity, Ionic Conductivity, and Self-Diffusion Coefficient) of Aprotic Heterocyclic Anion (AHA) Room Temperature Ionic Liquids. 2. Variation of Alkyl Chain Length in the Phosphonium Cation. J. Phys. Chem. B 2016, 120 (25), 5767–76. 10.1021/acs.jpcb.6b03934. 27243107

[ref75] Margulis C. J.; Annapureddy H. V. R.; De Biase P. M.; Coker D.; Kohanoff J.; Del Pópolo M. G. Dry Excess Electrons in Room-Temperature Ionic Liquids. J. Am. Chem. Soc. 2011, 133 (50), 20186–20193. 10.1021/ja203412v. 22032301

[ref76] Hapiot P.; Lagrost C. Electrochemical Reactivity in Room-Temperature Ionic Liquids. Chem. Rev. 2008, 108 (7), 2238–2264. 10.1021/cr0680686. 18564878

[ref77] Jacquemin J.; Husson P.; Padua A. A. H.; Majer V. Density and viscosity of several pure and water-saturated ionic liquids. Green Chem. 2006, 8 (2), 172–180. 10.1039/B513231B.

[ref78] Tariq M.; Carvalho P. J.; Coutinho J. A. P.; Marrucho I. M.; Lopes J. N. C.; Rebelo L. P. N. Viscosity of (C2-C14) 1-alkyl-3-methylimidazolium bis(trifluoromethylsulfonyl)amide ionic liquids in an extended temperature range. Fluid Phase Equilib. 2011, 301 (1), 22–32. 10.1016/j.fluid.2010.10.018.

[ref79] Rilo E.; Vila J.; García M.; Varela L. M.; Cabeza O. Viscosity and Electrical Conductivity of Binary Mixtures of CnMIM-BF4 with Ethanol at 288 K, 298 K, 308 K, and 318 K. J. Chem. Eng. Data 2010, 55 (11), 5156–5163. 10.1021/je100687x.

[ref80] Liao C.; Shao N.; Han K. S.; Sun X. G.; Jiang D. E.; Hagaman E. W.; Dai S. Physicochemical properties of imidazolium-derived ionic liquids with different C-2 substitutions. Phys. Chem. Chem. Phys. 2011, 13 (48), 21503–10. 10.1039/c1cp22375e. 22068150

[ref81] Xu W.; Wang L.-M.; Nieman R. A.; Angell C. A. Ionic Liquids of Chelated Orthoborates as Model Ionic Glassformers. J. Phys. Chem. B 2003, 107 (42), 11749–11756. 10.1021/jp034548e.

[ref82] Amith W. D.; Araque J. C.; Margulis C. J. A Pictorial View of Viscosity in Ionic Liquids and the Link to Nanostructural Heterogeneity. J. Phys. Chem. Lett. 2020, 11 (6), 2062–2066. 10.1021/acs.jpclett.0c00170. 32079397

[ref83] Harmon J.; Coffman C.; Villarrial S.; Chabolla S.; Heisel K. A.; Krishnan V. V. Determination of Molecular Self-Diffusion Coefficients Using Pulsed-Field-Gradient NMR: An Experiment for Undergraduate Physical Chemistry Laboratory. J. Chem. Educ. 2012, 89 (6), 780–783. 10.1021/ed200471k.

[ref84] Harris K. R.; Kanakubo M. Self-Diffusion Coefficients and Related Transport Properties for a Number of Fragile Ionic Liquids. J. Chem. Eng. Data 2016, 61 (7), 2399–2411. 10.1021/acs.jced.6b00021.

[ref85] Ue M.; Murakami A.; Nakamura S. A Convenient Method to Estimate Ion Size for Electrolyte Materials Design. J. Electrochem. Soc. 2002, 149 (10), A1385 10.1149/1.1507593.

[ref86] Nordness O.; Brennecke J. F. Ion Dissociation in Ionic Liquids and Ionic Liquid Solutions. Chem. Rev. 2020, 120 (23), 12873–12902. 10.1021/acs.chemrev.0c00373. 33026798

[ref87] Koddermann T.; Ludwig R.; Paschek D. On the validity of Stokes-Einstein and Stokes-Einstein-Debye relations in ionic liquids and ionic-liquid mixtures. ChemPhysChem 2008, 9 (13), 1851–8. 10.1002/cphc.200800102. 18752221

[ref88] Tokuda H.; Hayamizu K.; Watanabe M.; Ishii K.; Susan M. A. B. H. Physiochemical properties and structures of room temperature ionic liquids. 1. Variation of anionic species. J. Phys. Chem. B 2004, 108 (42), 16593 10.1021/jp047480r.

[ref89] Zhang H.; Yang L.; Fang S.; Peng C.; Luo H. Ionic liquids based on S-alkylthiolanium cations and TFSI anion as potential electrolytes. Chin. Sci. Bull. 2009, 54 (8), 1322–1327. 10.1007/s11434-009-0038-1.

[ref90] MacFarlane D. R.; Forsyth M.; Howlett P. C.; Pringle J. M.; Sun J.; Annat G.; Neil W.; Izgorodina E. I. Ionic Liquids in Electrochemical Devices and Processes: Managing Interfacial Electrochemistry. Acc. Chem. Res. 2007, 40 (11), 1165–1173. 10.1021/ar7000952. 17941700

[ref91] Webber A.; Blomgren G. E. Ionic Liquids for Lithium Ion and Related Batteries. In Advances in Lithium-Ion Batteries*;* van Schalkwijk W. A., Scrosati B., Eds.; Springer US: Boston, MA, 2002; pp 185–232.

[ref92] Yu L.; Chen G. Z. Ionic Liquid-Based Electrolytes for Supercapacitor and Supercapattery. Front. Chem. 2019, 7, 272 10.3389/fchem.2019.00272. 31058143 PMC6482234

[ref93] Ortega P. F. R.; Santos G. A. d.; Trigueiro J. P. C.; Silva G. G.; Quintanal N.; Blanco C.; Lavall R. L.; Santamaría R. Insights on the Behavior of Imidazolium Ionic Liquids as Electrolytes in Carbon-Based Supercapacitors: An Applied Electrochemical Approach. J. Phys. Chem. C 2020, 124 (29), 15818–15830. 10.1021/acs.jpcc.0c04217.

[ref94] Salanne M. Ionic Liquids for Supercapacitor Applications*;* Cham: Springer International Publishing: Cham, Switzerland, 2018; pp 29–53. 10.1007/s41061-017-0150-7 28560657

[ref95] Díaz M.; Ortiz A.; Ortiz I. Progress in the use of ionic liquids as electrolyte membranes in fuel cells. J. Membr. Sci. 2014, 469, 379–396. 10.1016/j.memsci.2014.06.033.

[ref96] Shen X.; Sun B.; Yan F.; Zhao J.; Zhang F.; Wang S.; Zhu X.; Lee S. High-Performance Photoelectrochemical Cells from Ionic Liquid Electrolyte in Methyl-Terminated Silicon Nanowire Arrays. ACS Nano 2010, 4 (10), 5869–5876. 10.1021/nn101980x. 20873809

[ref97] Liao C.; Shao N.; Han K. S.; Sun X.-G.; Jiang D.-E.; Hagaman E. W.; Dai S. Physicochemical properties of imidazolium-derived ionic liquids with different C-2 substitutions. Phys. Chem. Chem. Phys. 2011, 13 (48), 21503–21510. 10.1039/c1cp22375e. 22068150

[ref98] Xu W.; Cooper E. I.; Angell C. A. Ionic Liquids: Ion Mobilities, Glass Temperatures, and Fragilities. J. Phys. Chem. B 2003, 107 (25), 6170–6178. 10.1021/jp0275894.

[ref99] Austen Angell C.; Ansari Y.; Zhao Z. Ionic Liquids: Past, present and future. Faraday Discuss. 2012, 154 (0), 9–27. 10.1039/C1FD00112D. 22455011

[ref100] Bourlinos A. B.; Raman K.; Herrera R.; Zhang Q.; Archer L. A.; Giannelis E. P. A Liquid Derivative of 12-Tungstophosphoric Acid with Unusually High Conductivity. J. Am. Chem. Soc. 2004, 126 (47), 15358–15359. 10.1021/ja046821b. 15563144

[ref101] Fraser K. J.; Izgorodina E. I.; Forsyth M.; Scott J. L.; MacFarlane D. R. Liquids intermediate between “molecular” and “ionic” liquids: Liquid Ion Pairs?. Chem. Commun. 2007, (37), 3817–3819. 10.1039/b710014k. 18217657

[ref102] Nordness O.; Brennecke J. F. Ion Dissociation in Ionic Liquids and Ionic Liquid Solutions. Chem. Rev. 2020, 120 (23), 12873–12902. 10.1021/acs.chemrev.0c00373. 33026798

[ref103] Harris K. R. On the Use of the Angell-Walden Equation To Determine the “Ionicity” of Molten Salts and Ionic Liquids. J. Phys. Chem. B 2019, 123 (32), 7014–7023. 10.1021/acs.jpcb.9b04443. 31318219

[ref104] Shimizu K.; Tariq M.; Freitas A. A.; Pádua A. l. A. H.; Lopes J. N. C. Self-Organization in Ionic Liquids: From Bulk to Interfaces and Films. J. Braz. Chem. Soc. 2015, 27 (2), 349–362. 10.5935/0103-5053.20150274.

[ref105] Lewandowski A.; Świderska A. Electrochemical capacitors with polymer electrolytes based on ionic liquids. Solid State Ionics 2003, 161 (3), 243–249. 10.1016/S0167-2738(03)00275-3.

[ref106] Bard A. J. Electrochemical methods: fundamentals and applications*,* 2nd ed.; Bard A. J., Faulkner L. R., Eds.; John Wiley: New York, 2001.

[ref107] Voroshylova I. V.; Ers H.; Koverga V.; Docampo-Álvarez B.; Pikma P.; Ivaništšev V. B.; Cordeiro M. N. D. S. Ionic liquid-metal interface: The origins of capacitance peaks. Electrochim. Acta 2021, 379, 138148 10.1016/j.electacta.2021.138148.

[ref108] Vericat C.; Vela M. E.; Benitez G.; Carro P.; Salvarezza R. C. Self-assembled monolayers of thiols and dithiols on gold: new challenges for a well-known system. Chem. Soc. Rev. 2010, 39 (5), 1805–1834. 10.1039/b907301a. 20419220

[ref109] Grahame D. C. The Role of the Cation in the Electrical Double Layer. J. Electrochem. Soc. 1951, 98 (9), 343 10.1149/1.2778217.

[ref110] Waegele M. M.; Gunathunge C. M.; Li J.; Li X. How cations affect the electric double layer and the rates and selectivity of electrocatalytic processes. J. Chem. Phys. 2019, 151 (16), 160902 10.1063/1.5124878. 31675864

[ref111] Palchowdhury S.; Bhargava B. L. Surface Structure and Dynamics of Ions at the Liquid-Vapor Interface of Binary Ionic Liquid Mixtures: Molecular Dynamics Studies. J. Phys. Chem. C 2016, 120 (10), 5430–5441. 10.1021/acs.jpcc.5b10868. 26166036

[ref112] Wu B.; Yamashita Y.; Endo T.; Takahashi K.; Castner E. W. Jr. Structure and dynamics of ionic liquids: Trimethylsilylpropyl-substituted cations and bis(sulfonyl)amide anions. J. Chem. Phys. 2016, 145 (24), 244506 10.1063/1.4972410. 28049333

[ref113] Jasiecki S.; Serafińczuk J.; Gotszalk T.; Schroeder G. X-Ray Reflectometry Study of Self-Assembled Ionic Nanolayers. J. Nanomater. 2012, 2012, 568326 10.1155/2012/568326.

[ref114] Jang J. H.; Lydiatt F.; Lindsay R.; Baldelli S. Quantitative Orientation Analysis by Sum Frequency Generation in the Presence of Near-Resonant Background Signal: Acetonitrile on Rutile TiO2 (110). J. Phys. Chem. A 2013, 117 (29), 6288–6302. 10.1021/jp401019p. 23730957

[ref115] Pitawela N. R.; Shaw S. K. Capacitive Hysteresis Effects in Ionic Liquids: 1-Ethyl-3-methylimidazolium Trifluoromethanesulfonate on Polycrystalline Gold Electrode. J. Electrochem. Soc. 2021, 168 (4), 046510 10.1149/1945-7111/abf4ac.

[ref116] Vatamanu J.; Borodin O.; Bedrov D.; Smith G. Molecular Dynamics Simulation Study of the Interfacial Structure and Differential Capacitance of Alkylimidazolium Bis(trifluoromethanesulfonyl)imide [Cnmim][TFSI] Ionic Liquids at Graphite Electrodes. J. Phys. Chem. C 2012, 116, 7940–7951. 10.1021/jp301399b.

[ref117] Ji Y.; Shi R.; Wang Y.; Saielli G. Effect of the Chain Length on the Structure of Ionic Liquids: from Spatial Heterogeneity to Ionic Liquid Crystals. J. Phys. Chem. B 2013, 117 (4), 1104–1109. 10.1021/jp310231f. 23305509

[ref118] Wei K.; Deng L.; Wang Y.; Ou-Yang Z.-C.; Wang G. Effect of Side-Chain Length on Structural and Dynamic Properties of Ionic Liquids with Hydroxyl Cationic Tails. J. Phys. Chem. B 2014, 118 (13), 3642–3649. 10.1021/jp410168t. 24611796

[ref119] Klein J. M.; Squire H.; Gurkan B. Electroanalytical Investigation of the Electrode–Electrolyte Interface of Quaternary Ammonium Ionic Liquids: Impact of Alkyl Chain Length and Ether Functionality. J. Phys. Chem. C 2020, 124 (10), 5613–5623. 10.1021/acs.jpcc.9b08016.

[ref120] Georgi N.; Kornyshev A. A.; Fedorov M. V. The anatomy of the double layer and capacitance in ionic liquids with anisotropic ions: Electrostriction vs. lattice saturation. Journal of electroanalytical chemistry (Lausanne, Switzerland) 2010, 649 (1–2), 261–267. 10.1016/j.jelechem.2010.07.004.

[ref121] Fedorov M. V.; Georgi N.; Kornyshev A. A. Double layer in ionic liquids: The nature of the camel shape of capacitance. Electrochem. Commun. 2010, 12 (2), 296–299. 10.1016/j.elecom.2009.12.019.

[ref122] Zhao J.; Burke A. F. Review on supercapacitors: Technologies and performance evaluation. J. Energy Chem. 2021, 59, 276–291. 10.1016/j.jechem.2020.11.013.

[ref123] Mao X.; Brown P.; Červinka C.; Hazell G.; Li H.; Ren Y.; Chen D.; Atkin R.; Eastoe J.; Grillo I.; Padua A. A. H.; Costa Gomes M. F.; Hatton T. A. Self-assembled nanostructures in ionic liquids facilitate charge storage at electrified interfaces. Nat. Mater. 2019, 18 (12), 1350–1357. 10.1038/s41563-019-0449-6. 31406367

[ref124] Frontana-Uribe B. A.; Little R. D.; Ibanez J. G.; Palma A.; Vasquez-Medrano R. Organic electrosynthesis: a promising green methodology in organic chemistry. Green Chem. 2010, 12 (12), 2099–2119. 10.1039/c0gc00382d.

[ref125] Tiago G. a. A. O.; Matias I. A. S.; Ribeiro A. P. C.; Martins L. s. M. D. R. S. Application of Ionic Liquids in Electrochemistry-Recent Advances. Molecules 2020, 25 (24), 5812 10.3390/molecules25245812. 33317199 PMC7763911

