# Supplementary material for: Imidazolium Triflate Ionic Liquids’ Capacitance–Potential Relationships and Transport Properties Affected by Cation Chain Lengths
Source: ACS Meas Sci Au. 2021 Aug 10;1(3):117–30. doi: 10.1021/acsmeasuresciau.1c00015 (PMC9885949; doi:10.1021/acsmeasuresciau.1c00015)
Supplement: Supplementary file 1 — tg1c00015_si_001.pdf [file tg1c00015_si_001.pdf]

Supporting Information

TITLE

Imidazolium triflate Ionic Liquids' capacitance-potential relationships and transport properties affected by cation chain lengths

AUTHORS

Niroodha R. Pitawela and Scott K. Shaw*

Department of Chemistry, University of Iowa, Iowa City, Iowa 52242, United States

SI 1: The names of the selected ILs accompanied with their potential windows and water contents which are determined in triplicate using an 831 coulometric Karl Fischer (KF) titrator with a volume of 300 μL of IL.

SI 2: Calculation of electroactive surface area of the gold working electrode using the Randles-Sevcik equation.

SI 3: Equivalent circuit used to model experimental impedance data.

SI 4: Illustration of single frequency data analysis

SI 5: Determination of Ionic Liquid purity via ^{1}H NMR measurements.

SI 6 -10: The ^{1}H NMR chemical shifts of IL samples used.

SI 11: The average experimental viscosity data of five independent measurements of ILs studied measured at 25 °C and 60 °C.

SI I2: The average conductivity of each IL is determined using five independent conductivity measurements via the SevenCompact Conductivity meter S230-Std-Kit with a Mettler-Toledo 2-Cell conductivity electrode at 25 °C and 60 °C.

SI 13: The computed diffusion coefficients via Diffusion ordered NMR spectroscopy experiments (DOSY).

SI 14: Maximum capacitance calculated for single frequency impedance and AC voltammetry techniques using two different scanning protocols.

SI 15: AC Voltammetry data for five different ILs ([Emim][TFO], [Bmim][TFO], [Hmim][TFO], [Omim][TFO] and [Dmim][TFO]) on polycrystalline gold electrode obtained in
A) anodic direction
B) cathodic direction.
The arrow denotes the potential scan direction, and the arrows are numbered to indicate which scan direction is performed first. Anodic direction is performed first followed by cathodic direction. Error bars represent standard deviation of three replicate measurements under the same experimental conditions.

SI 16: Single frequency impedance data for five different ILs ([Emim][TFO], [Bmim][TFO], [Hmim][TFO], [Omim][TFO] and [Dmim][TFO]) on polycrystalline gold electrode obtained in
A) cathodic direction
B) anodic direction
The arrow denotes the potential scan direction, and the arrows are numbered to indicate which scan direction is performed first. Cathodic direction is performed first followed by anodic direction. Error bars represent standard deviation of three replicate measurements under the same experimental conditions.

SI 17: AC Voltammetry data for five different ILs ([Emim][TFO], [Bmim][TFO], [Hmim][TFO], [Omim][TFO] and [Dmim][TFO]) on polycrystalline gold electrode obtained in
A) cathodic direction
B) anodic direction
The arrow denotes the potential scan sequence. Cathodic direction is performed first followed by anodic direction. Error bars represent standard deviation of three replicate measurements under the same experimental conditions.

SI 18: Energy values calculated for single frequency impedance (SFI) and AC voltammetry (ACV) techniques using two different scanning protocols in J/L.

Ionic Liquid	Potential window (V)	Double layer region (V)	Literature data	Water content (ppm)
[Emim] [TFO]	3.6 V	2.0 V	4.1 V with Pt WE[1,2]<br>4.3 V with polycrystalline Au WE[3]	(3.0 ± 0.7) x 10^2
[Bmim][TFO]	4.0 V	3.0 V	3.5 V with polycrystalline Au WE[4]	(4.2 ± 0.7) x 10^2
[Hmim][TFO]	4.0 V	2.2 V	Data not reported	(6.3 ± 1.7) x 10^2
[Omim][TFO]	5.0 V	1.8 V	Data not reported	(6.6 ± 0.9) x 10^2
[Dmim][TFO]	6.0 V	2.0 V	Data not reported	(2.5 ± 0.7) x 10^2

SI 1: The names of the selected ILs accompanied with their potential windows and water contents which are determined in triplicate using an 831 coulometric Karl Fischer (KF) titrator with a volume of 300 µL of IL.

1. Ohno, H., *Electrochemical aspects of ionic liquids / edited by Hiroyuki Ohno*. Hoboken, N.J. : Wiley: 2011.
2. Tiejun, M.; Kwo-Hsiung, Y.; Diana, F. W.; Jean, N., Ionic Liquid-Based Non-Aqueous Electrolytes for Nickel/Metal Hydride Batteries. *Batteries* **2017,** *3* (1), 4.
3. Small, L.; Wheeler, D., Influence of Analysis Method on the Experimentally Observed Capacitance at the Gold-Ionic Liquid Interface. *J. Electrochem. Soc.* **2014,** *161* (4), H260-H263.
4. Gore, T. R.; Bond, T.; Zhang, W.; Scott, R. W. J.; Burgess, I. J., Hysteresis in the measurement of double-layer capacitance at the gold–ionic liquid interface. *Electrochemistry Communications* **2010,** *12* (10), 1340-1343.

The Randles-Sevcik equation [1] is used to calculate electroactive surface area:

$$\boldsymbol{i_p = 0.4463nFAC\,(\frac{nFvD}{RT})^{\frac{1}{2}}}$$

where i_p is the peak current, *n* is the number of electrons transferred; 1 for $Fe[CN_6]^{3-}$/ $Fe[CN_6]^{4-}$, *A* (cm^2) is the surface area of the electrode, *C* (mol cm^{-3}) is the concentration of the redox species.

SI 2: Calculation of electroactive surface area of the gold working electrode using the Randles-Sevcik equation

1. Bard, A. J., *Electrochemical methods : fundamentals and applications / Allen J. Bard, Larry R. Faulkner*. Second edition.. ed.; New York : John Wiley: 2001.

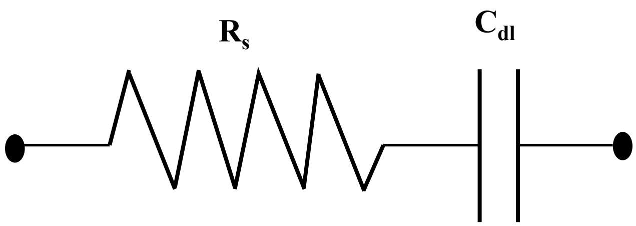

Equivalent circuit

$\mathbf{R_s}$= Solution Resistance

$\mathbf{C_{dl}}$ = Double Layer Capacitance

SI 3: Equivalent circuit used to model experimental impedance data

Single-frequency data analysis

The data analysis for single frequency impedance data is performed using Microsoft Excel and an example of the data analysis is given as a screenshot (Left side Figure). The generated data using the CH instruments 660D potentiostat are given in columns K to O in the screenshot, which represents the potential (E in volts), the real impedance (Z' in ohms), the imaginary impedance (Z" in ohms), the modulus of the impedance response (|Z| in ohms; shown as Z), and the phase angle (θ in degrees), respectively. The double layer capacitance is calculated using **Equation 2** and is given in micro-Farads in Excel cell P17; highlighted in yellow color. The function used to calculate the capacitance value is shown in the function box above the column headings. The double layer capacitance values are normalized with respect to the effective surface area of the gold working electrode and the calculated capacitance density values (see Right side Figure) are shown in Excel cell Q17, highlighted in green color.

P17 | fx =(-1/(M17*2*PI()*10))

	K	L	M	N	O	P	Q
1	Sept. 12		2020 17:44:39				
2	Impedance - Potential						
3	File: e:\research data\200912\20091		4ppm water tr 2 in	80v to 1	20v.csv		
4	Data Source: Experiment						
5	Instrument Model: CHI660D						
6	Header:						
7	Note:						
8							
9	Init E (V) = -0.8						
10	Final E (V) = 1.2						
11	Incr E (V) = 0.01						
12	Frequency (Hz) = 10						
13	Amplitude (V) = 0.01						
14	Quiet Time (sec) = 2						
15							
16	E (V)	Z' (Ω)	Z" (Ω)	Z (Ω)	θ (deg)	Capacitance (µF)	Capacitance density (µF cm^{-2})
17	-8.00E-01	1.35E+04	-3.23E+04	3.50E+04	-6.72E+01	4.94E-07	14.82
18	-7.90E-01	1.25E+04	-3.30E+04	3.53E+04	-6.92E+01	4.82E-07	14.48
19	-7.80E-01	1.16E+04	-3.35E+04	3.55E+04	-7.09E+01	4.75E-07	14.26
20	-7.70E-01	1.08E+04	-3.38E+04	3.55E+04	-7.23E+01	4.70E-07	14.13
21	-7.60E-01	1.01E+04	-3.40E+04	3.54E+04	-7.34E+01	4.69E-07	14.08
22	-7.50E-01	9.51E+03	-3.39E+04	3.52E+04	-7.43E+01	4.69E-07	14.09
23	-7.40E-01	9.00E+03	-3.38E+04	3.50E+04	-7.51E+01	4.71E-07	14.15
24	-7.30E-01	8.57E+03	-3.35E+04	3.46E+04	-7.57E+01	4.75E-07	14.26
25	-7.20E-01	8.20E+03	-3.32E+04	3.42E+04	-7.61E+01	4.79E-07	14.40
26	-7.10E-01	7.88E+03	-3.28E+04	3.38E+04	-7.65E+01	4.85E-07	14.57
27	-7.00E-01	7.61E+03	-3.24E+04	3.33E+04	-7.68E+01	4.91E-07	14.76
28	-6.90E-01	7.37E+03	-3.20E+04	3.28E+04	-7.70E+01	4.98E-07	14.96
29	-6.80E-01	7.16E+03	-3.15E+04	3.23E+04	-7.72E+01	5.05E-07	15.16
30	-6.70E-01	6.96E+03	-3.11E+04	3.19E+04	-7.74E+01	5.11E-07	15.36
31	-6.60E-01	6.79E+03	-3.08E+04	3.15E+04	-7.76E+01	5.17E-07	15.54
32	-6.50E-01	6.63E+03	-3.05E+04	3.12E+04	-7.77E+01	5.23E-07	15.69
33	-6.40E-01	6.49E+03	-3.02E+04	3.09E+04	-7.79E+01	5.27E-07	15.82
34	-6.30E-01	6.36E+03	-3.01E+04	3.07E+04	-7.81E+01	5.29E-07	15.90
35	-6.20E-01	6.24E+03	-3.00E+04	3.06E+04	-7.82E+01	5.31E-07	15.95
36	-6.10E-01	6.13E+03	-2.99E+04	3.05E+04	-7.84E+01	5.32E-07	15.98
37	-6.00E-01	6.03E+03	-2.99E+04	3.05E+04	-7.86E+01	5.32E-07	15.97

Q17 | fx =P17/(0.0333*10^-6)

	K	L	M	N	O	P	Q
1	Sept. 12		2020 17:44:39				
2	Impedance - Potential						
3	File: e:\research data\200912\20091		4ppm water tr 2 in	80v to 1	20v.csv		
4	Data Source: Experiment						
5	Instrument Model: CHI660D						
6	Header:						
7	Note:						
8							
9	Init E (V) = -0.8						
10	Final E (V) = 1.2						
11	Incr E (V) = 0.01						
12	Frequency (Hz) = 10						
13	Amplitude (V) = 0.01						
14	Quiet Time (sec) = 2						
15							
16	E (V)	Z' (Ω)	Z" (Ω)	Z (Ω)	θ (deg)	Capacitance (µF)	Capacitance density (µF cm^{-2})
17	-8.00E-01	1.35E+04	-3.23E+04	3.50E+04	-6.72E+01	4.94E-07	14.82
18	-7.90E-01	1.25E+04	-3.30E+04	3.53E+04	-6.92E+01	4.82E-07	14.48
19	-7.80E-01	1.16E+04	-3.35E+04	3.55E+04	-7.09E+01	4.75E-07	14.26
20	-7.70E-01	1.08E+04	-3.38E+04	3.55E+04	-7.23E+01	4.70E-07	14.13
21	-7.60E-01	1.01E+04	-3.40E+04	3.54E+04	-7.34E+01	4.69E-07	14.08
22	-7.50E-01	9.51E+03	-3.39E+04	3.52E+04	-7.43E+01	4.69E-07	14.09
23	-7.40E-01	9.00E+03	-3.38E+04	3.50E+04	-7.51E+01	4.71E-07	14.15
24	-7.30E-01	8.57E+03	-3.35E+04	3.46E+04	-7.57E+01	4.75E-07	14.26
25	-7.20E-01	8.20E+03	-3.32E+04	3.42E+04	-7.61E+01	4.79E-07	14.40
26	-7.10E-01	7.88E+03	-3.28E+04	3.38E+04	-7.65E+01	4.85E-07	14.57
27	-7.00E-01	7.61E+03	-3.24E+04	3.33E+04	-7.68E+01	4.91E-07	14.76
28	-6.90E-01	7.37E+03	-3.20E+04	3.28E+04	-7.70E+01	4.98E-07	14.96
29	-6.80E-01	7.16E+03	-3.15E+04	3.23E+04	-7.72E+01	5.05E-07	15.16
30	-6.70E-01	6.96E+03	-3.11E+04	3.19E+04	-7.74E+01	5.11E-07	15.36
31	-6.60E-01	6.79E+03	-3.08E+04	3.15E+04	-7.76E+01	5.17E-07	15.54
32	-6.50E-01	6.63E+03	-3.05E+04	3.12E+04	-7.77E+01	5.23E-07	15.69
33	-6.40E-01	6.49E+03	-3.02E+04	3.09E+04	-7.79E+01	5.27E-07	15.82
34	-6.30E-01	6.36E+03	-3.01E+04	3.07E+04	-7.81E+01	5.29E-07	15.90
35	-6.20E-01	6.24E+03	-3.00E+04	3.06E+04	-7.82E+01	5.31E-07	15.95
36	-6.10E-01	6.13E+03	-2.99E+04	3.05E+04	-7.84E+01	5.32E-07	15.98
37	-6.00E-01	6.03E+03	-2.99E+04	3.05E+04	-7.86E+01	5.32E-07	15.97

Example Excel spreadsheet demonstrating the calculation of double layer capacitance from single-frequency impedance data.

Example Excel spreadsheet demonstrating the calculation of capacitance density from single-frequency impedance data

SI 4: Illustration of single frequency data analysis

IL sample purity was analyzed by ^{1}H NMR measurements. ^{1}H NMR spectroscopy is a non-destructive technique and gives a complete analysis of possible organic impurities found in the IL. The typical impurities of ILs are water, halides, and alkyl imidazole starting reagents.[1] As discussed previously, the water content of an IL is an important parameter determining IL's electrochemical stability window. However, an increase in the water content affects the viscosity and conductivity of an IL system in a favorable way. Hence, we collect ^{1}H NMR measurements of Ionic Liquids (diluted in d-acetonitrile (CD_3CN)) to survey for water and other proton containing impurities. These data are shown in **SI Figures S2** to **S6**. A resonance at 2.13 ppm is assigned to water for the [Bmim][TFO] IL sample. In all the other ILs, the water peak was not visible, but it is possible that other resonances have masked the intensity of the water peak. NMR chemical shifts for each IL sample are given in tables underneath **SI Figures S2 to S6.** The chemical shift of solvent peak (CD_3CN) remains at the same position for all the ILs studied. Hence, this solvent peak is used to integrate the other ^{1}H NMR peaks as shown in **SI Figures S2 to S6**, to get the number of hydrogens in each environment. Ultimately, the ^{1}H NMR spectra are similar to those of pure ILs with very minor evidence of impurities, mitigating any concerns of significant impurity effects in this work.

SI 5: Determination of Ionic Liquid purity via ^{1}H NMR measurements

1. Lazarus, L. L.; Riche, C. T.; Malmstadt, N.; Brutchey, R. L., Effect of ionic liquid impurities on the synthesis of silver nanoparticles. *Langmuir* **2012,** *28* (45), 15987-93.

SI 6: 1H NMR spectrum of [Emim][TFO] in CD_3CN, recorded at 500 MHz. The respective chemical shifts and spin multiplicities for the interested peaks are listed in the table below. s, br s, q, t denote the singlet, broad singlet, quartet, and triplet, respectively.

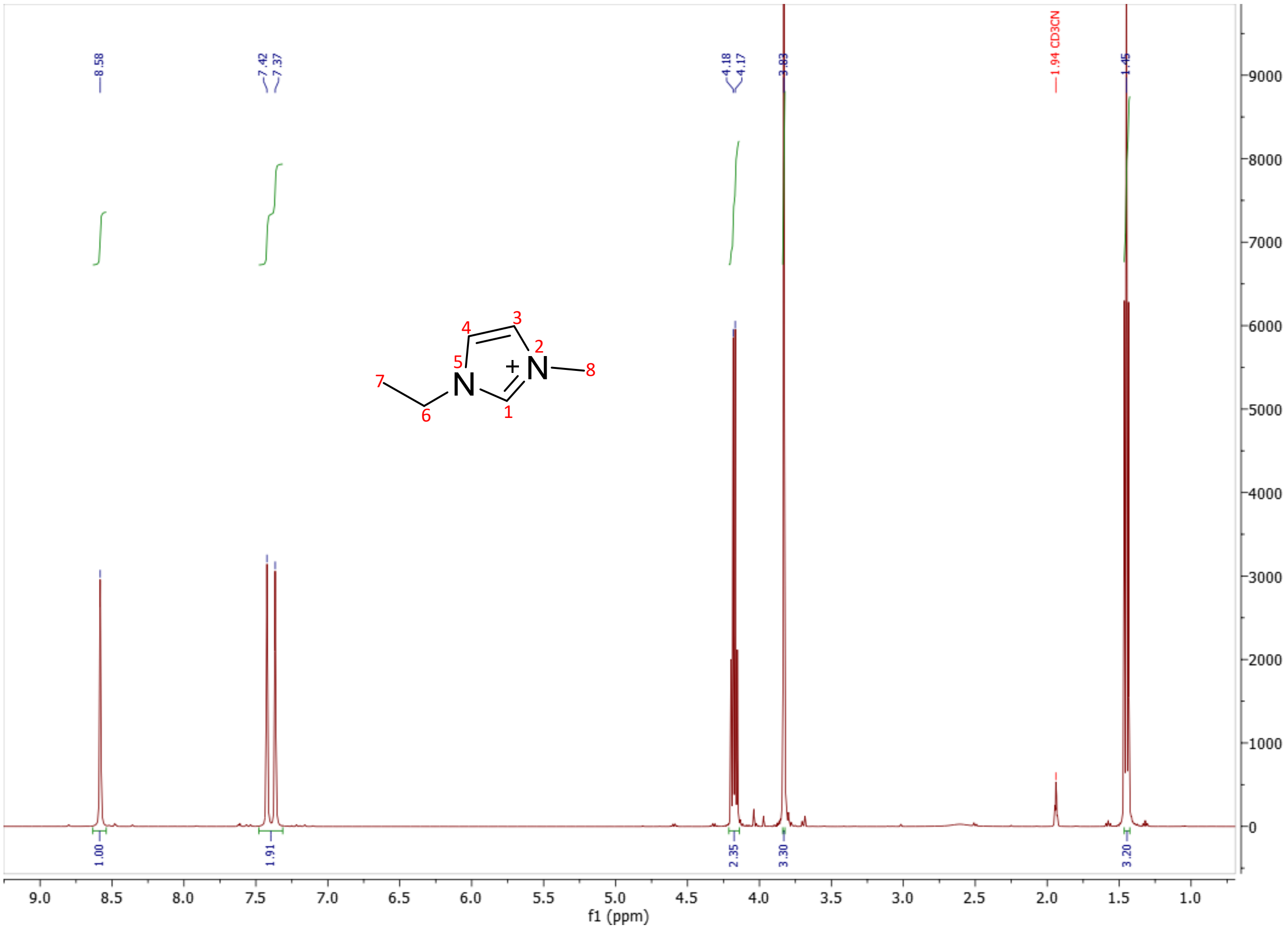

Position	δ_H (mult, J in Hz)[a]
1	8.58 (s)
3	7.37 (br s)
4	7.42 (br s)
6	4.18 (q, 7.5)
7	1.45 (t, 7.5)
8	3.83 (s)

SI 7: 1H NMR spectrum of [Bmim][TFO] in CD_3CN, recorded at 500 MHz. The respective chemical shifts and spin multiplicities for the interested peaks are listed in the table below. s, br s, q, t denote the singlet, broad singlet, quartet, and triplet, respectively.

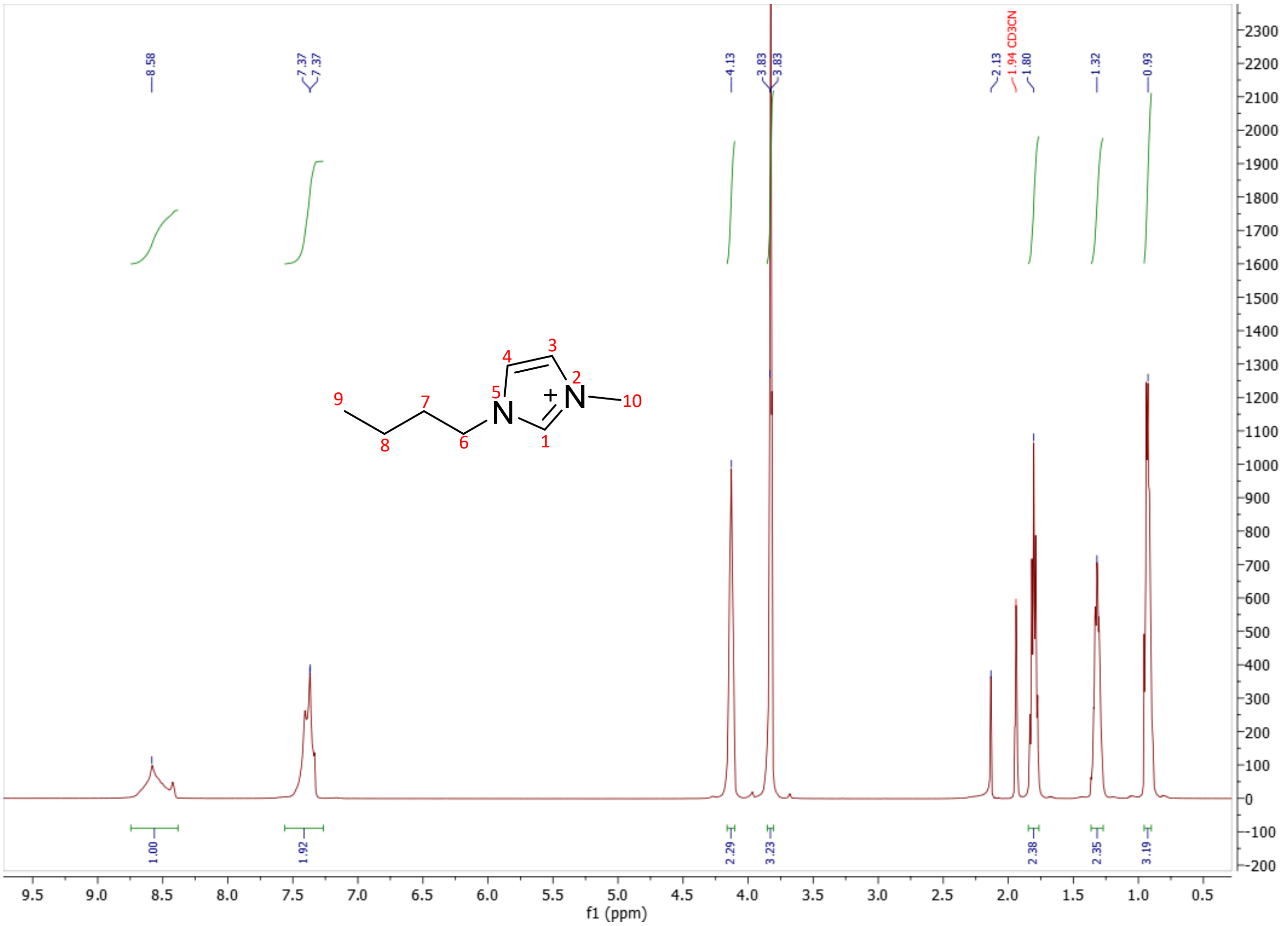

Position	δ_H (mult, J in Hz)[a]
1	8.58 (m)
3	7.41-7.33 (m)
4	7.41-7.33 (m)
6	4.14-4.11 (m)
7	1.80 (p, 7.6)
8	1.36-1.28 (m)
9	0.95-0.89 (m)
10	3.82 (br s)

Figure S3: 1H NMR spectrum of [Bmim][TFO. The respective chemical shifts and spin multiplicities for the interested peaks are listed in the table below. s, br s, q, t denote the singlet, broad singlet, quartet, and triplet respectively.

SI 8: 1H NMR spectrum of [Hmim][TFO] in CD_3CN, recorded at 500 MHz. The respective chemical shifts and spin multiplicities for the interested peaks are listed in the table below. s, br s, q, t denote the singlet, broad singlet, quartet, and triplet, respectively.

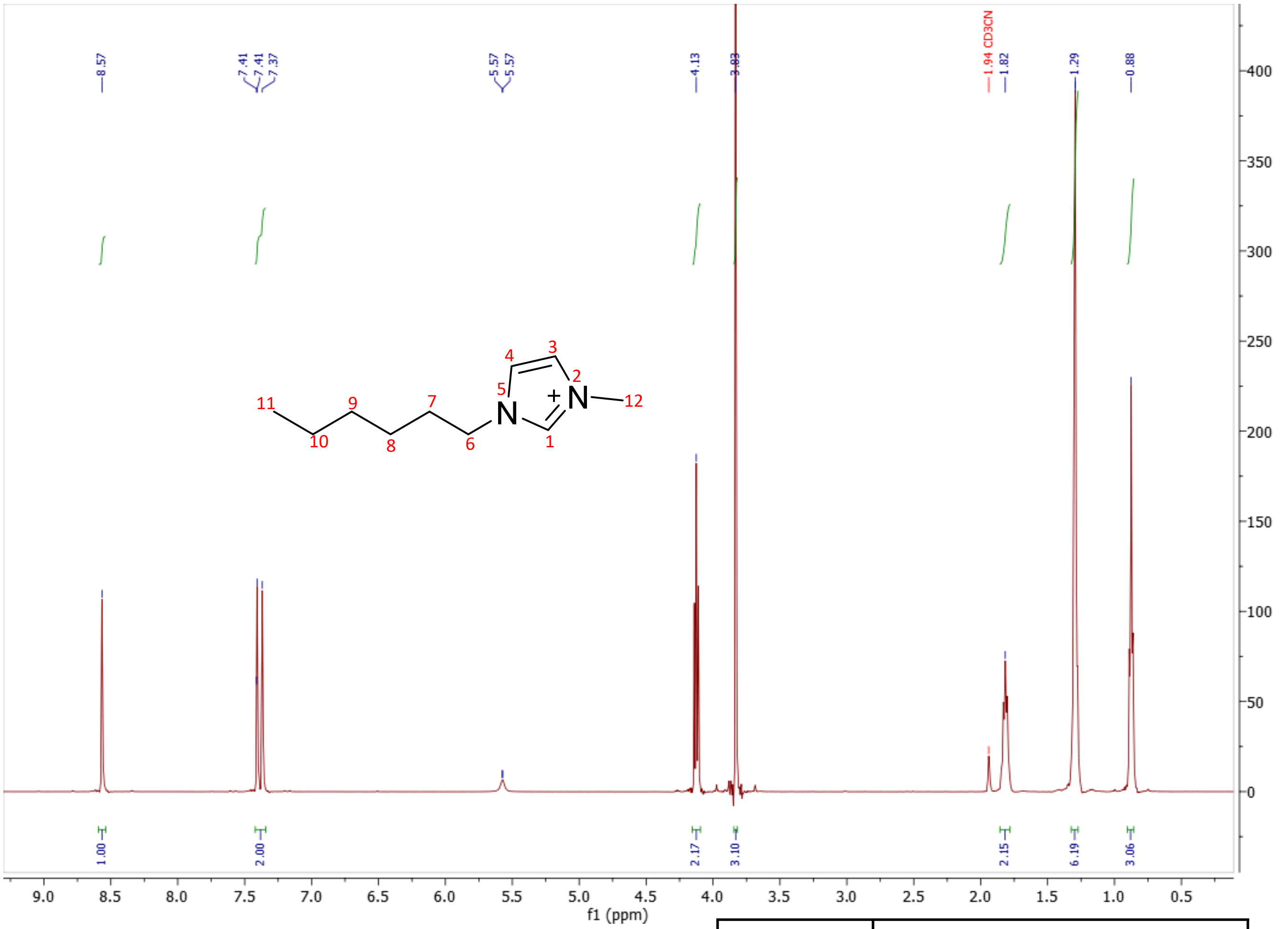

Position	δ_H (mult, J in Hz)[a]
1	8.57 (s)
3	7.37 (br s)
4	7.41 (br s)
6	4.12 (t, 7.2)
7	1.81 (p, 7.0)
8	1.32-1.27 (m)
9	1.32-1.27 (m)
10	1.32-1.27 (m)
11	0.87 (t, 6.8)
12	3.83 (s)

SI 9: 1H NMR spectrum of [Omim][TFO] in CD_3CN, recorded at 500 MHz. The respective chemical shifts and spin multiplicities for the interested peaks are listed in the table below. s, br s, q, t denote the singlet, broad singlet, quartet, and triplet, respectively.

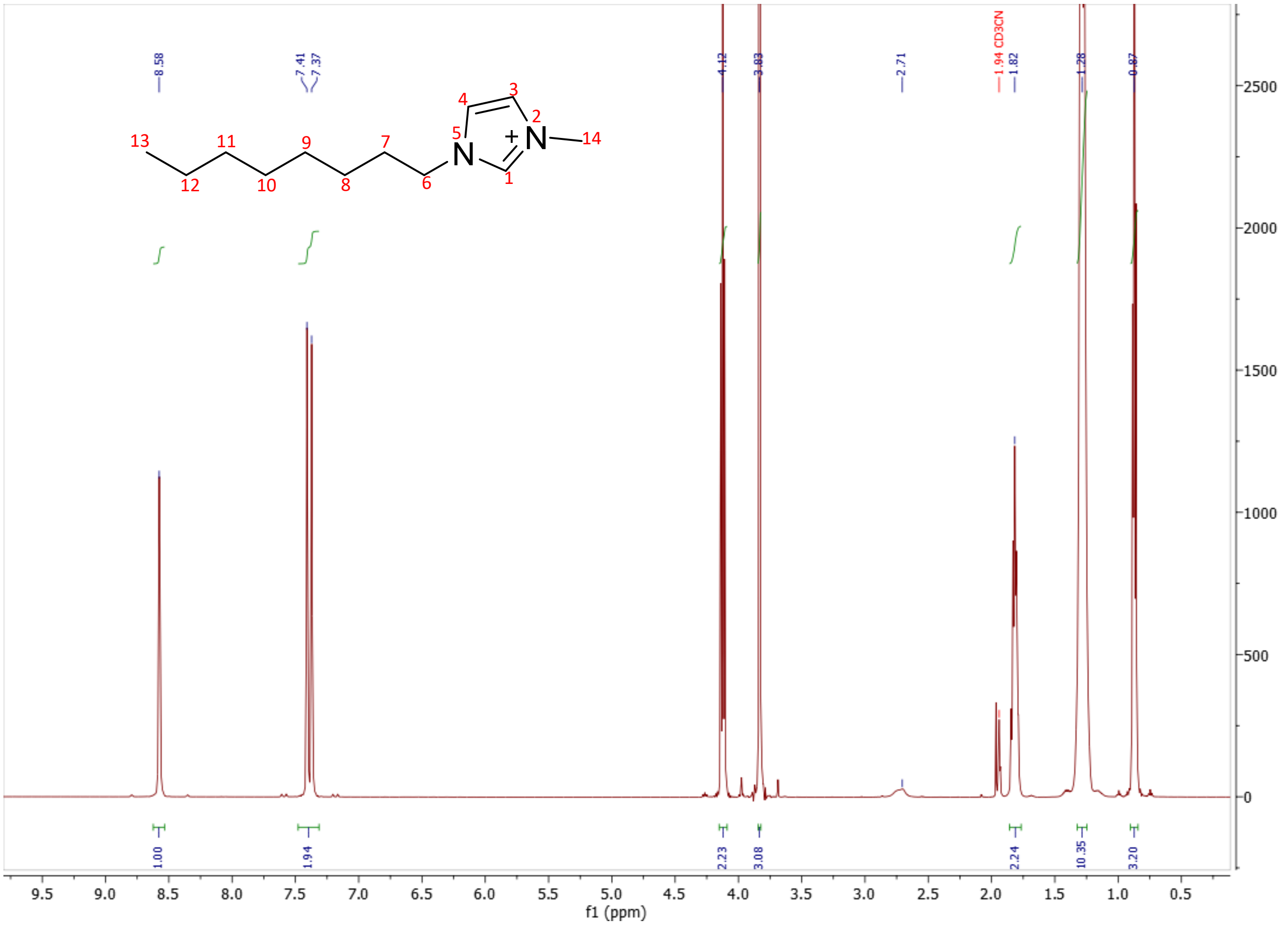

Position	δ_H (mult, J in Hz)[a]
1	8.58 (s)
3	7.41 (br s)
4	7.37 (br s)
6	4.12 (t, 7.3)
7	1.85-1.79 (m)
8	1.30-1.26 (m)
9	1.30-1.26 (m)
10	1.30-1.26 (m)
11	1.30-1.26 (m)
12	1.30-1.26 (m)
13	0.87 (t, 6.7)
14	3.83 (s)

SI 10: 1H NMR spectrum of [Dmim][TFO] in CD_3CN, recorded at 500 MHz. The respective chemical shifts and spin multiplicities for the interested peaks are listed in the table below. s, br s, q, t denote the singlet, broad singlet, quartet, and triplet, respectively.

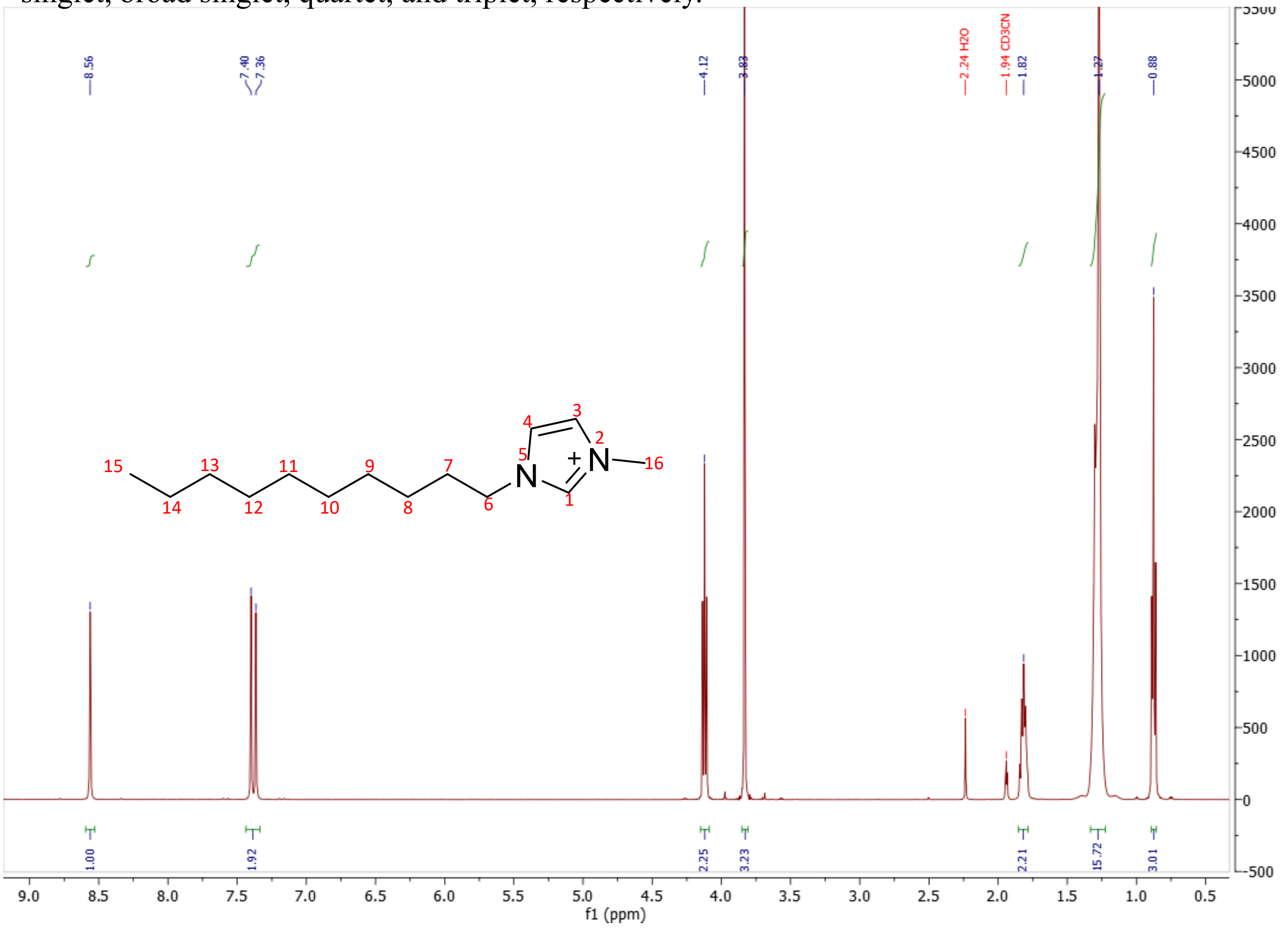

Position	δ_H (mult, J in Hz)[a]
1	8.56 (s)
3	7.40 (br s)
4	7.36 (br s)
6	4.12 (t, 7.34)
7	1.81 (p, 7.3)
8	1.30-1.27 (m)
9	1.30-1.27 (m)
10	1.30-1.27 (m)
11	1.30-1.27 (m)
12	1.30-1.27 (m)
13	1.30-1.27 (m)
14	1.30-1.27 (m)
15	0.87 (t, 7.1)
16	3.83 (s)

Ionic liquid	Viscosity at 25 °C this work (cP)	Literature Viscosity at 25 °C (cP)	Viscosity at 60 °C (cP) this work	Literature Viscosity at 60 °C (cP)
[Emim][TFO]	47 ± 1	45.9[1]	12± 1	Not published
[Bmim][TFO]	95 ± 8	90[2]	21 ± 0.4	Not published
[Hmim][TFO]	139 ± 4	135[3]	31 ± 1	Not published
[Omim][TFO]	182 ± 3	225[4]	37 ± 1	Not published
[Dmim][TFO]	296 ± 11	298[5]	52 ± 1	Not published

SI 11: Average and stdev experimental viscosity data of five independent measurements of ILs studied measured at 25 °C and 60 °C. Literature values are reported for comparison where available.

1. Mbondo Tsamba, B. E.; Sarraute, S.; Traïkia, M.; Husson, P., Transport Properties and Ionic Association in Pure Imidazolium-Based Ionic Liquids as a Function of Temperature. *Journal of Chemical & Engineering Data* **2014,** *59* (6), 1747-1754.
2. Hapiot, Philippe, & Lagrost, Corinne. (2008). Electrochemical Reactivity in Room-Temperature Ionic Liquids. *Chemical Reviews, 108*(7), 2238-2264.
3. https://iolitec.de/index.php/en/products/ionic_liquids/catalogue/imidazolium-based/il-0070-hp
4. https://iolitec.de/en/node/141
5. https://iolitec.de/en/node/72

Ionic liquid	Conductivity at 25 °C (mS/cm)	Literature value for Conductivity at 25 °C (mS/cm)	Conductivity at 60 °C (mS/cm)	Literature value for Conductivity at 60 °C (mS/cm)
[Emim][TFO]	7.44 ± 0.35	9.13[1]	21.31 ± 1.62	Not published
[Bmim][TFO]	2.29 ± 0.16	2.90[2]	11.17 ± 0.14	Not published
[Hmim][TFO]	0.94 ± 0.05	Not published	6.67 ± 0.29	Not published
[Omim][TFO]	0.57 ± 0.01	Not published	3.62 ± 0.06	Not published
[Dmim][TFO]	0.31 ± 0.06	Not published	2.31 ± 0.01	Not published

SI 12: The average conductivity of each IL is determined using five independent conductivity measurements via the SevenCompact Conductivity meter S230-Std-Kit with a Mettler-Toledo 2-Cell conductivity electrode at 25 °C and 60 °C.

1. Mbondo Tsamba, B. E, Sarraute, S, Traïkia, M, & Husson, P. (2014). Transport Properties and Ionic Association in Pure Imidazolium-Based Ionic Liquids as a Function of Temperature. *Journal of Chemical and Engineering Data, 59*(6), 1747-1754.
2. Hapiot, Philippe, & Lagrost, Corinne. (2008). Electrochemical Reactivity in Room-Temperature Ionic Liquids. *Chemical Reviews, 108*(7), 2238-2264

Ionic Liquid	Cation diffusion coefficient at 25 °C (cm^2/s)	Cation diffusion coefficient at 25 °C (cm^2/s): (Literature)	Cation diffusion coefficient at 60 °C (cm^2/s):	Anion diffusion coefficient at 25 °C (cm^2/s):	Anion diffusion coefficient at 25 °C (cm^2/s): (Literature)
[Emim][TFO]	4.3×10^{-7}	4.1×10^{-7}**	2.79×10^{-6}	2.5×10^{-7}	2.6×10^{-7}**
[Bmim][TFO]	1.8×10^{-7}	1.7×10^{-7}**	1.61×10^{-6}	-	1.3×10^{-7}**
[Hmim][TFO]	1.1×10^{-7}	-	1.27×10^{-6}	-	-
[Omim][TFO]	6.1×10^{-8}	-	1.10×10^{-6}	-	-
[Dmim][TFO]	*	-	8.00×10^{-7}	-	-

SI 13: The computed diffusion coefficients via Diffusion ordered NMR spectroscopy experiments (DOSY) (*) out of the measurement range of the present work. Limited by the sensitivity of the NMR probe.

** Mbondo Tsamba, B. E.; Sarraute, S.; Traïkia, M.; Husson, P., Transport Properties and Ionic Association in Pure Imidazolium-Based Ionic Liquids as a Function of Temperature. *Journal of Chemical & Engineering Data* **2014,** *59* (6), 1747-1754.

Ionic liquid	SFI Maximum Capacitance (µF cm^{-2})				ACV Maximum Capacitance (µF cm^{-2})			
	Anodic to Cathodic		Cathodic to Anodic		Anodic to Cathodic		Cathodic to Anodic	
	Anodic	Cathodic	Cathodic	Anodic	Anodic	Cathodic	Cathodic	Anodic
[Emim][TFO]	17.8 ± 0.3	23.9 ± 0.2	22.7 ± 0.1	16.9 ± 0.4	11.4 ± 0.9	15.4 ± 0.4	14 ± 1	11.2 ± 0.1
[Bmim][TFO]	21.3 ± 0.9	24 ± 1	23.4 ± 0.2	20.4 ± 0.4	14.1 ± 0.5	19 ± 1	17.8 ± 0.6	15 ± 2
[Hmim][TFO]	33 ± 1	39 ± 1	50 ± 7	45 ± 6	21 ± 1	25 ± 1	33 ± 3	32 ± 3
[Omim][TFO]	26 ± 2	32 ± 2	27.9 ± 0.1	22.7 ± 0.3	16 ± 2	21.4 ± 0.4	18.4 ± 0.1	15.3 ± 0.1
[Dmim][TFO]	42 ± 4	48 ± 3	39 ± 2	30 ± 2	28 ± 1	34 ± 2	28 ± 4	21 ± 2

SI 14: Maximum capacitance calculated for single frequency impedance (SFI) and AC voltammetry (ACV) techniques using two different scanning protocols.

The capacitance values obtained using the single-frequency impedance potential technique are treated using the ***Equation 3***;

$$\boldsymbol{C_{dl} = \frac{-1}{Z'' \cdot 2\pi f}} \quad \textbf{\textit{Equation 3}}$$

Here f is the applied AC frequency in hertz, and Z″ is the imaginary impedance.[1-6]

AC voltammetry is also a single frequency impedance technique, and its data are converted into double layer capacitance (C_{dl}) via ***Equation 4***:

$$\boldsymbol{C = \frac{I''}{\omega \cdot \Delta E_{amp}}\left[1 + \left(\frac{I'}{I''}\right)^2\right]} \quad \textbf{\textit{Equation 4}}$$

where I′ and I″ are the in- and out-of-phase currents, $\omega = 2\pi f$, and ΔE_{amp} is the perturbation amplitude respectively.[7]

1. Copper, C. L.; Whitaker, K. W., Capillary Electrophoresis: Part II. Applications. *Journal of Chemical Education* **1998,** *75* (3), 347.
2. Lucio, A. J.; Shaw, S. K., Effects and controls of capacitive hysteresis in ionic liquid electrochemical measurements. *Analyst* **2018,** *143* (20), 4887-4900.
3. Nanjundiah, C.; McDevitt, S.; Koch, V., Differential capacitance measurements in solvent-free ionic liquids at Hg and C interfaces. *Journal of the Electrochemical Society (USA)* **1997,** *144* (10), 3392-3397.
4. Small, L.; Wheeler, D., Influence of Analysis Method on the Experimentally Observed Capacitance at the Gold-Ionic Liquid Interface. *J. Electrochem. Soc.* **2014,** *161* (4), H260-H263.
5. Alam, M. T.; Mominul Islam, M.; Okajima, T.; Ohsaka, T., Measurements of differential capacitance in room temperature ionic liquid at mercury, glassy carbon and gold electrode interfaces. *Electrochemistry Communications* **2007,** *9* (9), 2370-2374.
6. Alam, M. T.; Masud, J.; Islam, M. M.; Okajima, T.; Ohsaka, T., Differential Capacitance at Au(111) in 1-Alkyl-3-methylimidazolium Tetrafluoroborate Based Room-Temperature Ionic Liquids. *The Journal of Physical Chemistry C* **2011,** *115* (40), 19797-19804.
7. Gore, T. R.; Bond, T.; Zhang, W.; Scott, R. W. J.; Burgess, I. J., Hysteresis in the measurement of double-layer capacitance at the gold–ionic liquid interface. *Electrochemistry Communications* **2010,** *12* (10), 1340-1343.

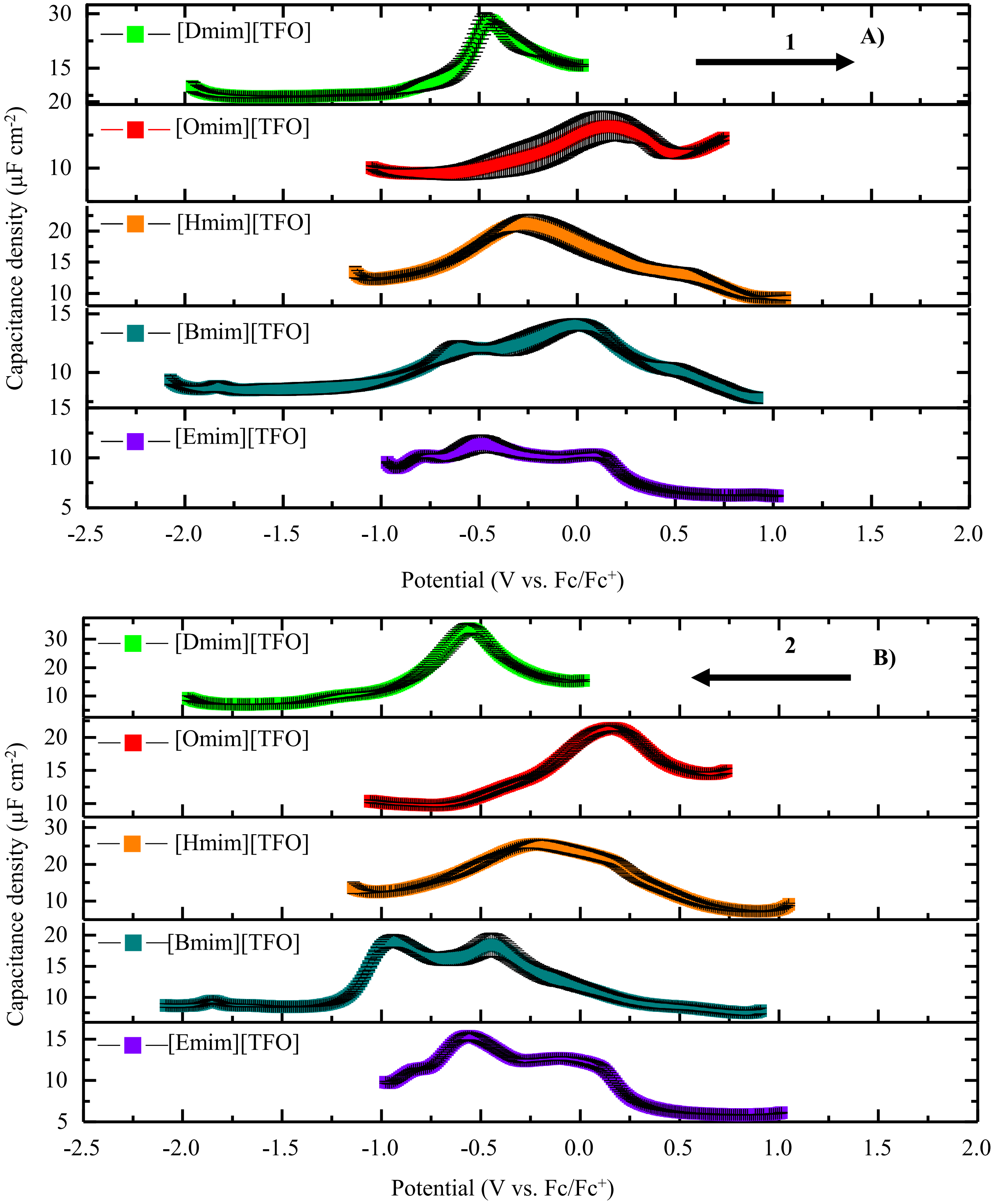

SI 15: AC Voltammetry data for five different ILs ([Emim][TFO], [Bmim][TFO], [Hmim][TFO], [Omim][TFO] and [Dmim][TFO]) on polycrystalline gold electrode obtained in

A) anodic direction

B) cathodic direction.

The arrow denotes the potential scan direction, and the arrows are numbered to indicate which scan direction is performed first. Anodic direction is performed first followed by cathodic direction. Error bars represent standard deviation of three replicate measurements under the same experimental conditions.

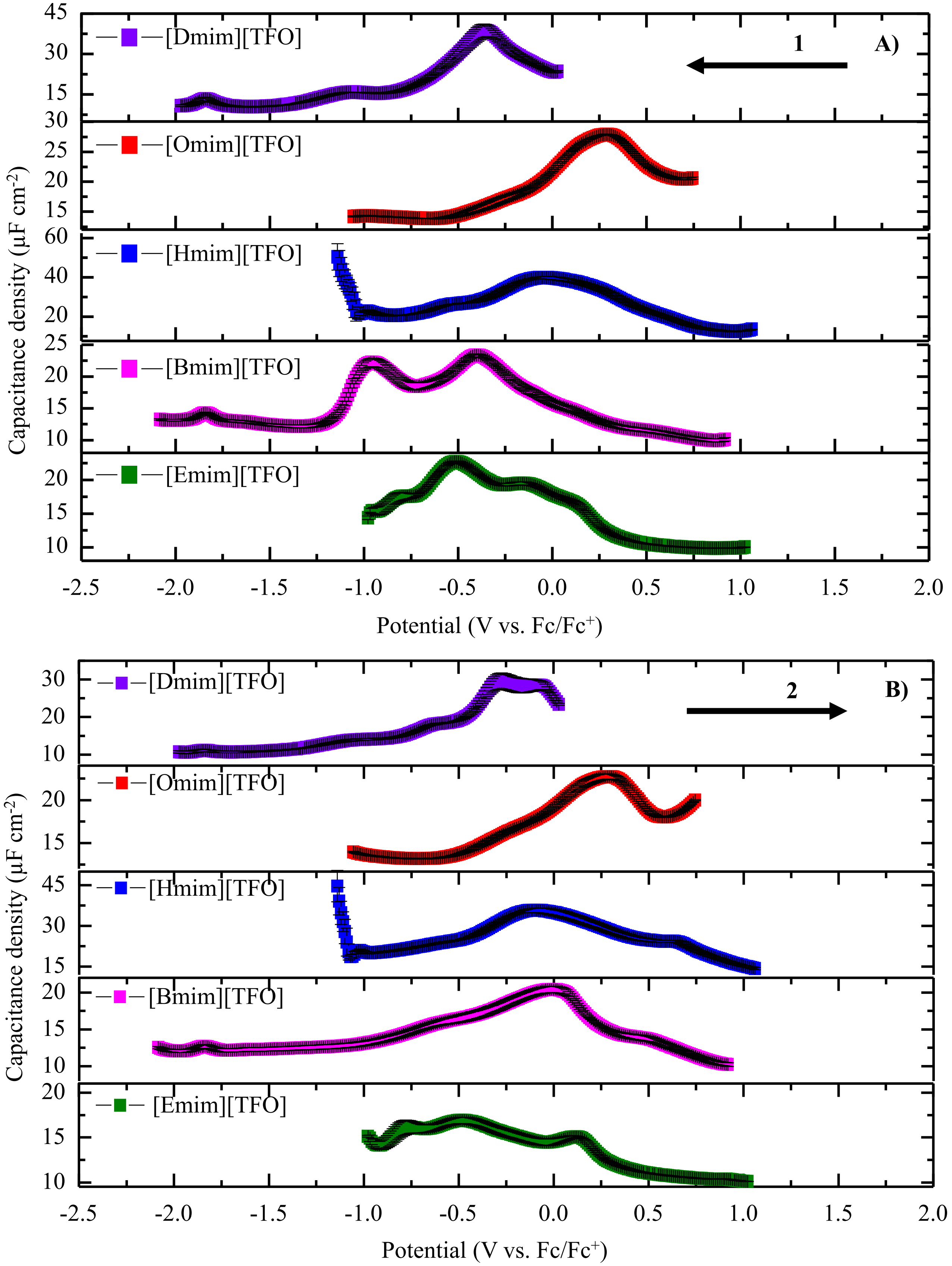

SI 16: Single frequency impedance data for five different ILs ([Emim][TFO], [Bmim][TFO], [Hmim][TFO], [Omim][TFO] and [Dmim][TFO]) on polycrystalline gold electrode obtained in

A) cathodic direction

B) anodic direction

The arrow denotes the potential scan direction, and the arrows are numbered to indicate which scan direction is performed first. Cathodic direction is performed first followed by anodic direction. Error bars represent standard deviation of three replicate measurements under the same experimental conditions.

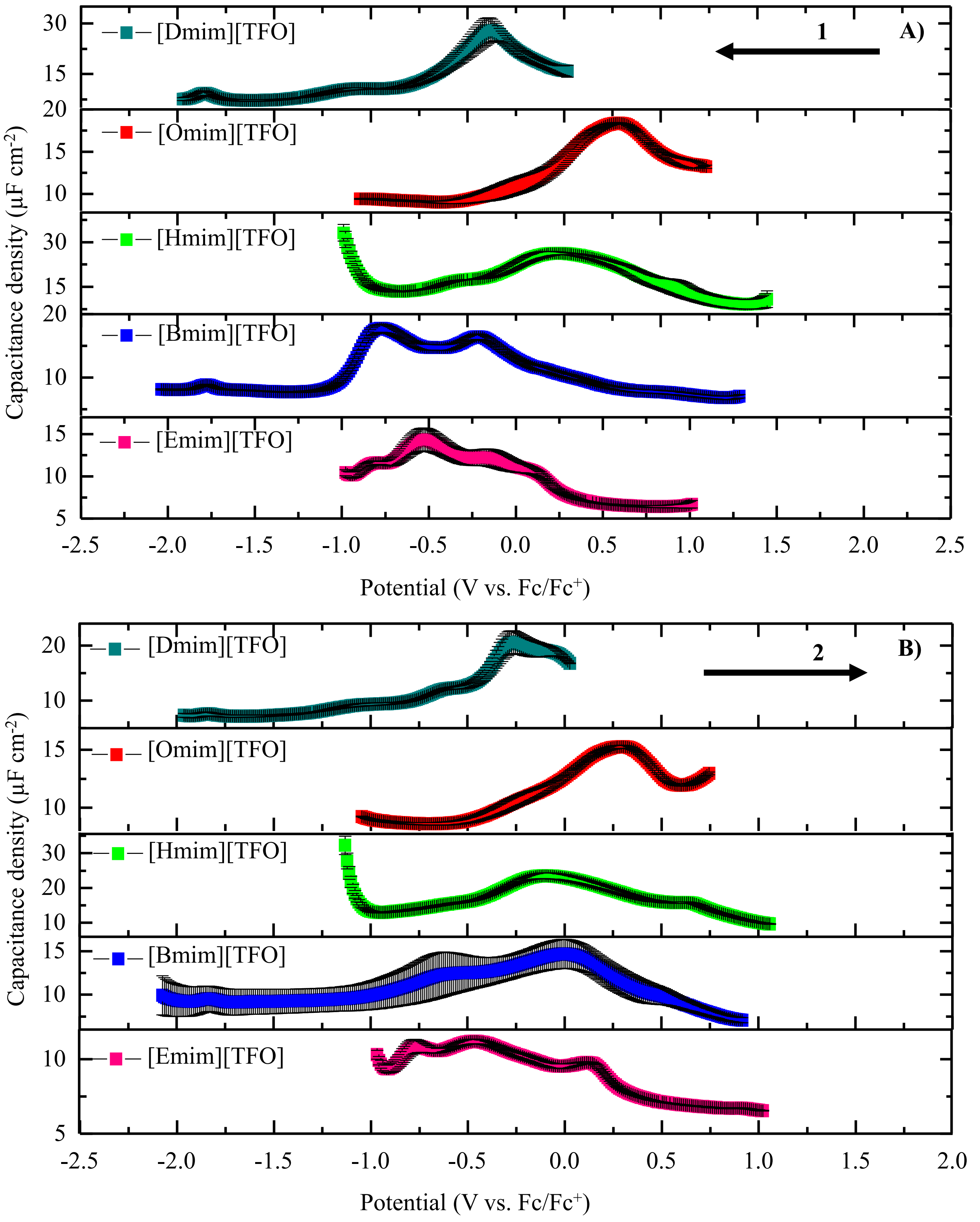

SI 17: AC Voltammetry data for five different ILs ([Emim][TFO], [Bmim][TFO], [Hmim][TFO], [Omim][TFO] and [Dmim][TFO]) on polycrystalline gold electrode obtained in

A) cathodic direction

B) anodic direction

The arrow denotes the potential scan sequence. Cathodic direction is performed first followed by anodic direction. Error bars represent standard deviation of three replicate measurements under the same experimental conditions.

Ionic liquid	SFI Energy x 10^3 (J/L) ± percent relative uncertainty				ACV Energy x 10^3 (J/L) ± percent relative uncertainty			
	Anodic to Cathodic		Cathodic to Anodic		Anodic to cathodic		Cathodic to Anodic	
	Anodic	Cathodic	Cathodic	Anodic	Anodic	Cathodic	Cathodic	Anodic
[Emim][TFO]	1079 ± 2	1448 ± 1	1376 ± 1	1024 ± 2	691 ± 8	933 ± 3	848 ± 9	679 ± 1
[Bmim][TFO]	2905 ± 4	3273 ± 4	3191 ± 1	2782 ± 2	1923 ± 3	2591 ± 5	2427 ± 4	2050 ± 10
[Hmim][TFO]	2420 ± 4	2860 ± 2	3670 ± 1	3300 ± 10	1540 ± 7	1833 ± 4	2420 ± 9	2347 ± 8
[Omim][TFO]	1276 ± 6	1571 ± 3	1370 ± 1	1114 ± 1	790 ± 10	1051 ± 2	903 ± 1	751 ± 1
[Dmim][TFO]	2545 ± 9	2909 ± 7	2364 ± 6	1818 ± 7	1697 ± 4	2061 ± 5	1700 ± 10	1273 ± 10

SI 18: Tabulated **e**nergy density (given in units J/L) for the IL systems studies here, listed for measurement technique (SFI= single frequency impedance, and ACV=alternating current voltammetry) and direction of the applied potential sweep. Energy density (E) is calculated using; $E = (CV^2)/2$ where C is the maximum capacitance and V is the double layer region potential window. Error propagation is performed only for the maximum capacitance value as an average of n ≥ 3 independent measurements. Calculated uncertainties are shown with the energy density values.

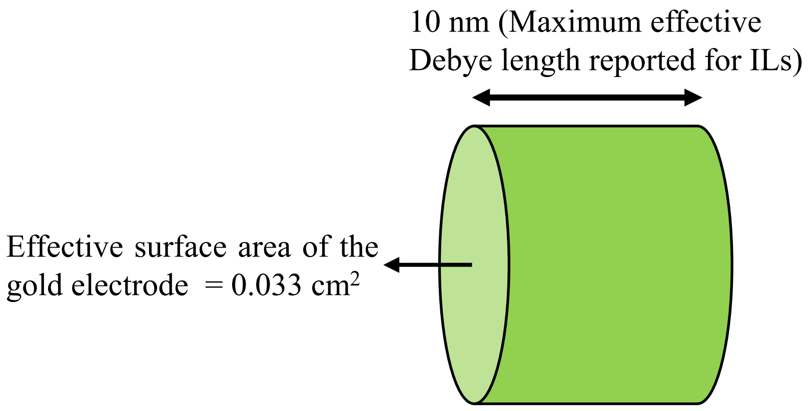

Volume of the Ionic Liquid in contact with the gold working electrode $= area\ x\ height = 0.033\ cm^2\ x\ 10\ nm = 3.3\ x\ 10^{-11}\ Liters$

Energy = [capacitance x $(\text{voltage})^2$]/ 2

$$\text{Energy density stored in (J/L)} = \frac{Energy\ in\ J}{Volume\ of\ Ionic\ Liquid\ in\ contact\ with\ the\ gold\ working\ electrode\ (L)}$$
